# Supplementary material for: Short-term effectiveness of nutrition therapy to treat type 2 diabetes in low-income and middle-income countries: systematic review and meta-analysis of randomised controlled trials
Source: BMJ Open. 2022 Mar 10;12(3):e056108. doi: 10.1136/bmjopen-2021-056108 (PMC8915303; doi:10.1136/bmjopen-2021-056108)
Supplement: Supplementary data [file bmjopen-2021-056108supp001.pdf]

SUPPLEMENTARY MATERIAL 1

MEDLINE search strategy and number of results at each stage

1	diabetes mellitus/ or exp diabetes mellitus, type 2/ or exp hyperglycemia/ or exp metabolic syndrome/ (273807)
2	(diabet* adj5 treat*).tw. (64270)
3	(diabet* adj5 diagnos*).tw. (24777)
4	1 or 2 or 3 (322372)
5	exp Diet/ (261138)
6	*nutrition therapy/ or exp diet therapy/ (52679)
7	exp Nutrition Policy/ (9911)
8	exp nutrition assessment/ (13903)
9	nutrition disorders/ or exp overnutrition/ (213134)
10	((health* or wellness or weight or diet*) adj2 (treat* or Interven* or change* or improve*)).tw. (147274)
11	exp Food Labeling/ (3392)
12	5 or 6 or 7 or 8 or 9 or 10 or 11 (576981)
13	(afghan* or angola* or bangladesh* or benin* or bhutan* or "burkina fasso" or burundi* or cambodia* or "central african republic" or chad* or comoros or congo* or "democratic republic of the congo" or Djibouti* or eritrea* or ethiopia* or gambia* or guinea* or guinea-bissau or haiti* or kiribati* or "lao people's democratic republic" or lao or lesotho* or liberia* or madagasca* or malawi* or mali* or mauritania* or mozambique or myanmar or nepal* or niger or rwanda* or "sao tome and principe" or senegal* or "sierra leone" or "solomon islands" or somalia* or "south sudan" or sudan* or tanzania* or "timor-leste" or togo or tuvalu* or uganda* or vanuatu or yemen or zambia*).tw. (792163)
14	(armenia* or bolivia* or "cabo verde" or "cape verde" or cameroon* or congo* or "cote d'ivoire" or egypt* or "el salvador" or eswatini or georgia* or ghana* or guatemala* or honduras or india* or indonesia* or jordan* or kenya* or kosov* or kyrgyzstan or micronesia* or moldova* or mogolia* or morocc* or nicaragua* or nigeria* or pakistan* or "papua new guinea" or philippin* or "sri lanka" or "syrian arab republic" or syria* or tajikistan or tokelau or tunisia* or Ukrain* or uzbekistan or "viet nam" or "west bank and gaza strip" or "democratic people's republic of korea" or zimbabwe*).tw. (345506)
15	(albania* or algeria* or "antigua and barbuda" or argentin* or azerbaijan or belarus or belize or "bosnia and herzegovina" or botswana* or brazil* or china or colombia* or "cook islands" or "costa rica" or cuba* or dominica* or "dominican republic" or ecuador* or "equatorial guinea" or fiji or "former yugoslav republic of macedonia" or gabon or grenada or guyana* or iran* or iraq* or jamaica* or kazakhstan or lebanon or libya or Malaysia or Maldives or "marshall islands" or Mauritius or Mexico or Montenegro or Montserrat or namibia* or nauru or niue or palau or panama or paraguay or peru* or "saint helena" or "saint lucia" or "saint vincent and the grenadines" or samoa* or serbia* or "south africa" or suriname or thailand or tonga* or turkey or turkmenistan or venezuela* or "wallis and futuna").tw. (507391)
16	developing country.mp. or Developing Countries/ (75649)
17	((developing or less* developed or under developed or underdeveloped or middle income or low* income or deprived or poor) adj (countr* or nation or population)).tw. (75342)
18	13 or 14 or 15 or 16 or 17 (1632917)
19	randomi* controlled trial.tw. (81488)
20	controlled clinical trial.pt. (93000)
21	randomized controlled trial.pt. (479114)

22	random*.ab. (988614)
23	trial.ab. (448719)
24	19 or 20 or 21 or 22 or 23 (1388842)
25	4 and 12 and 18 and 24 (630)
