# Supplementary material for: Short-term effectiveness of nutrition therapy to treat type 2 diabetes in low-income and middle-income countries: systematic review and meta-analysis of randomised controlled trials
Source: BMJ Open. 2022 Mar 10;12(3):e056108. doi: 10.1136/bmjopen-2021-056108 (PMC8915303; doi:10.1136/bmjopen-2021-056108)
Supplement: Supplementary data [file bmjopen-2021-056108supp002.pdf]

SUPPLEMENTARY MATERIAL 2

Where included studies differed in measuring units used for glycated hemoglobin (HbA1c), fasting blood glucose, and other biomarkers these were converted to enable comparison. The following formulas were applied:

Fasting Blood Glucose (Table 1): Three studies reported fasting blood glucose in milligrams per deciliter (mg/dL), whereas one study[1] measured fasting blood glucose in millimoles per liter (mmol/L). The formula applied for conversion was: mg/dL = 18.01*mmol/l [2,3].

	Baseline M(SD)		Follow-up (6 months post-intervention) M(SD)	
	**Original (mmol/L)**	**Converted (mg/dl)**	**Original (mmol/L)**	**Converted (mg/dl)**
**Experimental Intervention**	8.0 (3.9)	160.2 (70.2)	8.1 (2.7)	145.8 (48.6)
**Control intervention**	8.3 (2.9)	149.4 (52.2)	7.8 (2.6)	136.8 (46.8)

Table 1: Unit transformation for Fasting Blood Glucose (Ramadas[1]). M: Mean, SD: Standard Deviation

HDL and LDL lipoprotein-cholesterol (Tables 2 and 3): The two studies that included biomarkers of diabetes complications used different units. Therefore, values in mmol/l were converted to mg/dl using the following formula: mg/dL = 38.67*mmol/l [4]

	Baseline M(SD)		Follow-up (6 months post-intervention) M(SE)	
	**Original (mmol/L)**	**Converted (mg/dl)**	**Original (mmol/L)**	**Converted (mg/dl)**
**Experimental Intervention**	3.00 (0.90)	116.67 (34.80)	2.66 (0.08)	103.74(3.12)
**Control intervention**	3.10(0.90)	119.88 (34.80)	2.74 (0.08)	106.86 (3.12)

Table 2: Unit transformation for LDL lipoprotein-cholesterol(Muchiri[5]); M: Mean, SD: Standard Deviation, SE: Standard Error

	Baseline M(SD)		Follow-up (6 months post-intervention) M(SE)	
	**Original (mmol/L)**	**Converted (mg/dl)**	**Original (mmol/L)**	**Converted (mg/dl)**
**Experimental Intervention**	1.10 (0.27)	42.54 (10.44)	0.99 (0.02)	38.61 (0.78)

**Control intervention**	1.10 (0.27)	42.54 (10.44)	1.0 (0.03)	39 (1.17)

Table 3: Unit transformation for HDL lipoprotein-cholesterol (Muchiri[5]); M: Mean, SD: Standard deviation, SE: Standard Error

Triglycerides (Table 4): The two studies that included biomarkers of diabetes complications used different units. Therefore, values in mmol/l were converted to mg/dl using the following formula: mg/dL = 88.57*mmol/l[4].

	**Baseline M(CI)**		**Follow-up (6 months post-intervention) M(CI)**	
	**Original (mmol/L)**	**Converted (mg/dl)**	**Original (mmol/L)**	**Converted (mg/dl)**
**Experimental Intervention**	1.40 (1.10 to 1.60)	124 (97.43 to 141.71)	1.30 (1.20 to 1.60)	115.7 (97.90 to 142.40)
**Control intervention**	1.40 (1.20 to 1.60)	124 (106.28 to 141.71)	1.50 (1.30 to 1.80)	133.5 (115.70 to 160.20)

Table 4: Unit transformation for triglycerides (Muchiri[5]); M: Mean, CI: 95% Confidence Interval

Standard Error/Standard Deviation (Tables 5-11): reporting by Muchiri[5] included standard deviation for baseline values, but standard error for follow-up values.

	**Baseline**		**Follow-up (6 months post-intervention)**	
	**Original** M(SD)	**Converted** M(SD)	**Original** M(SE)	**Converted** M(SD)
**Experimental Intervention**	10.80 (1.80)	NA	9.67 (0.29)	9.67 (1.86)
**Control intervention**	11.40 (2.20)	NA	10.3 (0.29)	10.3 (1.9)

Table 5: Transformation from Standard Error to Standard Deviation at follow-up measures of HbA1c. NA = Not applicable.

	**Baseline**		**Follow-up (6 months post-intervention)**	
	**Original** M(SD)	**Converted** M(SD)	**Original** M(SE)	**Converted** M(SD)
**Experimental Intervention**	31.50 (7.00)	NA	30.50 (0.20)	30.5 (1.30)
**Control intervention**	30.40 (6.80)	NA	30.80 (0.20)	30.80 (1.28)

Table 6: Transformation from Standard Error to Standard Deviation at follow-up measures of BMI. NA = Not applicable.

	Baseline		Follow-up (6 months post-intervention)	
	**Original** M(SD)	**Converted** M(SD)	**Original** M(SE)	**Converted** M(SD)
**Experimental Intervention**	3.0 (0.90)	NA	103.74(3.12)	103.74(19.98)
**Control intervention**	3.1 (0.90)	NA	106.86 (3.12)	106.86 (19.98)

Table 7: Transformation from Standard Error to Standard Deviation at follow-up measures of LDL-Cholesterol. NA = Not applicable.

	Baseline		Follow-up (6 months post-intervention)	
	**Original** M(SD)	**Converted** M(SD)	**Original** M(SE)	**Converted** M(SD)
**Experimental Intervention**	3.0 (0.90)	NA	38.61 (0.78)	38.61 (4.99)
**Control intervention**	3.10 (0.90)	NA	39 (1.17)	39 (7.49)

Table 8: Transformation from Standard Error to Standard Deviation at follow-up measures of HDL-Cholesterol. NA = Not applicable.

	Baseline		Follow-up (6 months post-intervention)	
	**Original** M(SD)	**Converted** M(SD)	**Original** M(SE)	**Converted** M(SD)
**Experimental Intervention**	1.40 (1.10 to 1.60)	NA	115.7 (97.90 to 142.40)	115.7 (72.69)
**Control intervention**	1.40 (1.20 to 1.60)	NA	133.5 (115.70 to 160.20)	133.5 (72.69)

Table 9: Transformation from Confidence Interval (CI to Standard Deviation at follow-up measures of triglycerides. NA = Not applicable.

	Baseline		Follow-up (6 months post-intervention)	
	**Original** M(SD)	**Converted** M(SD)	**Original** M(SE)	**Converted** M(SD)
**Experimental Intervention**	142.9(22.9)	NA	134.1 (2.9)	134.1 (18.57)
**Control intervention**	143.3 (28)	NA	130.1 (2.9)	130.1 (18.57)

Table 10: Transformation from Standard Error to Standard Deviation at follow-up measures of systolic blood pressure. NA = Not applicable.

	Baseline		Follow-up (6 months post-intervention)	
	**Original** M(SD)	**Converted** M(SD)	**Original** M(SE)	**Converted** M(SD)

**Experimental Intervention**	84.3 (11.7)	NA	78.9 (1.5)	78.9 (9.60)
**Control intervention**	84.5 (11.7)	NA	78.8 (1.5)	78.8 (9.60)

Table 11: Transformation from Standard Error to Standard Deviation at follow-up measures of diastolic blood pressure. NA = Not applicable.

References

1 Ramadas A, Chan CKY, Oldenburg B, *et al.* Randomised-controlled trial of a web-based dietary intervention for patients with type 2 diabetes: Changes in health cognitions and glycemic control. *BMC Public Health* 2018;**18**:1–13. doi:10.1186/s12889-018-5640-1

2 Somogyi M. STUDIES OF ARTERIOVENOUS DIFFERENCES IN BLOOD SUGAR. *J Biol Chem* 1948;**174**:189–200. doi:10.1016/S0021-9258(18)57386-5

3 American Diabetes Association. Glycemic targets. *Diabetes Care* 2015;**38**:S33–40. doi:10.2337/dc15-S009

4 Rugge B, Balshem H, Sehgal R, *et al.* Lipid Conversion Factors. Published Online First: 2011.https://www.ncbi.nlm.nih.gov/books/NBK83505/ (accessed 13 May 2021).

5 Muchiri JW, Gericke GJ, Rheeder P. Effect of a nutrition education programme on clinical status and dietary behaviours of adults with type 2 diabetes in a resource-limited setting in South Africa: A randomised controlled trial. *Public Health Nutr* 2016;**19**:142–55. doi:10.1017/S1368980015000956
