# Supplementary material for: Short-term effectiveness of nutrition therapy to treat type 2 diabetes in low-income and middle-income countries: systematic review and meta-analysis of randomised controlled trials
Source: BMJ Open. 2022 Mar 10;12(3):e056108. doi: 10.1136/bmjopen-2021-056108 (PMC8915303; doi:10.1136/bmjopen-2021-056108)
Supplement: Author's manuscript [file bmjopen-2021-056108.draft_revisions.pdf]

BMJ Open

BMJ Open is committed to open peer review. As part of this commitment we make the peer review history of every article we publish publicly available.

When an article is published we post the peer reviewers' comments and the authors' responses online. We also post the versions of the paper that were used during peer review. These are the versions that the peer review comments apply to.

The versions of the paper that follow are the versions that were submitted during the peer review process. They are not the versions of record or the final published versions. They should not be cited or distributed as the published version of this manuscript.

BMJ Open is an open access journal and the full, final, typeset and author-corrected version of record of the manuscript is available on our site with no access controls, subscription charges or pay-per-view fees (http://bmjopen.bmj.com).

If you have any questions on BMJ Open's open peer review process please email info.bmjopen@bmj.com

BMJ Open

Short-term effectiveness of nutritional therapy to treat type 2 diabetes in low- and middle-income countries: systematic review of randomized-controlled trials.

Journal:	*BMJ Open*
Manuscript ID	bmjopen-2021-056108
Article Type:	Original research
Date Submitted by the Author:	06-Aug-2021
Complete List of Authors:	Guilbert, Elizabeth; University of Bristol, Faculty of Health Sciences<br>Perry, Rachel; NIHR Bristol Biomedical Research Centre<br>Whitmarsh, Alex; NIHR Bristol Biomedical Research Centre<br>Sauchelli, Sarah; NIHR Bristol Biomedical Research Centre
Keywords:	General diabetes < DIABETES & ENDOCRINOLOGY, NUTRITION & DIETETICS, PUBLIC HEALTH

SCHOLARONE™
Manuscripts

I, the Submitting Author has the right to grant and does grant on behalf of all authors of the Work (as defined in the below author licence), an exclusive licence and/or a non-exclusive licence for contributions from authors who are: i) UK Crown employees; ii) where BMJ has agreed a CC-BY licence shall apply, and/or iii) in accordance with the terms applicable for US Federal Government officers or employees acting as part of their official duties; on a worldwide, perpetual, irrevocable, royalty-free basis to BMJ Publishing Group Ltd ("BMJ") its licensees and where the relevant Journal is co-owned by BMJ to the co-owners of the Journal, to publish the Work in this journal and any other BMJ products and to exploit all rights, as set out in our licence.

The Submitting Author accepts and understands that any supply made under these terms is made by BMJ to the Submitting Author unless you are acting as an employee on behalf of your employer or a postgraduate student of an affiliated institution which is paying any applicable article publishing charge ("APC") for Open Access articles. Where the Submitting Author wishes to make the Work available on an Open Access basis (and intends to pay the relevant APC), the terms of reuse of such Open Access shall be governed by a Creative Commons licence – details of these licences and which Creative Commons licence will apply to this Work are set out in our licence referred to above.

Other than as permitted in any relevant BMJ Author's Self Archiving Policies, I confirm this Work has not been accepted for publication elsewhere, is not being considered for publication elsewhere and does not duplicate material already published. I confirm all authors consent to publication of this Work and authorise the granting of this licence.

For peer review only

Short-term effectiveness of nutritional therapy to treat type 2 diabetes in low- and middle-income countries: systematic review of randomized-controlled trials.

*Elizabeth Guilbert[1,2], Rachel Perry[3], Alex Whitmarsh[3], Sarah Sauchelli[3]**

[1] *Queen Elizabeth University Hospital, NHS Greater Glasgow and Clyde*
[2]*Faculty of Health Sciences, University of Bristol, Bristol, United Kingdom*
[3] *National Institute for Health Research Bristol Biomedical Research Centre, University Hospitals of Bristol and Weston NHS Foundation Trust and University of Bristol, Bristol, United Kingdom.*

Corresponding author:
Sarah Sauchelli
National Institute for Health Research Bristol Biomedical Research Centre, University Hospitals of Bristol and Weston NHS Foundation Trust and University of Bristol, Bristol, United Kingdom.
ORCID iD: orcid.org/0000-0003-3620-7671
Phone: 01173421758
Email: sarah.sauchellitoran@bristol.ac.uk

Word count: 3639
Keyword: type 2 diabetes, nutritional therapy, systematic review, LMIC

For peer review only

ABSTRACT

Introduction

Type 2 diabetes mellitus (T2DM) is a global pandemic, with 79% of individuals affected living in low- and middle-income countries (LMICs). Nutritional therapy could provide sustainable, economically efficient methods for improving glycaemic control and preventing medical complications. This review examined the evidence arising from randomized controlled trials regarding the impact of nutritional therapy on glycaemic control in people living with T2DM in LMICs.

Methods

MEDLINE, EMBASE, Web of Science, OpenGrey and the International Clinical Trials Registry Platform were searched. Randomized controlled trials were included if they evaluated nutritional therapy conducted in LMICs, measured glycaemic control and the trial included at least a 3-month, post-intervention assessment. 2 independent reviewers screened the database. Study characteristics and outcome data were extracted using a data collection form. Trials were assessed for risk of bias and GRADE certainty of evidence.

Results

4 trials met inclusion criteria, conducted in Malaysia, Iran, and South Africa, focusing on nutrition education with a maximum duration of 8 weeks. Compared to standard care, the implementation of nutrition education did not meaningfully impact levels of glycated haemoglobin (mean difference (MD): -0.63%, 95% CI -1.46 to 0.21) or fasting blood glucose (MD: -0.63%, 95% CI -1.46 to 0.21). Given the small number of eligible trials, moderate to high risk of publication bias and serious concerns regarding consistency and precision of the evidence, certainty of evidence was deemed to be very low.

Conclusion

Existing randomized controlled trials with a 3-month follow-up assessment suggest that nutritional therapy alone does not improve glycaemic control in people living with T2DM in LMICs. More comprehensive lifestyle interventions may be required. There is a lack of well-conducted randomized controlled trials that examine the long-term impact of nutritional therapy. Further research is essential to discover realistic, evidence-based treatment solutions in the care of T2DM patients in LMICs.

For peer review only

Article Summary: Strengths and limitations of this study

- This is the first synthesis of evidence for the effectiveness of nutritional therapy for the management of type 2 diabetes mellitus in low- to income-countries derived from randomised controlled trials that have included follow-up assessment.
- The review was conducted following BMJ Best Practice research methods, such as the use of GRADE (Grading of Recommendations, Assessment, Development and Evaluations) to provide a transparent critical appraisal of the certainty of evidence.
- The identification of few studies that met eligibility criteria highlight the need to build capacity for high-quality and long-term research to guide context appropriate evidence-based medicine, and to consider pragmatic trials as alternative research approaches to gather evidence.
- The focus on randomized controlled trials that included a follow-up assessment signifies that research on other interventions that adopted a different study design were not captured.

For peer review only

For peer review only - http://bmjopen.bmj.com/site/about/guidelines.xhtml

INTRODUCTION

Type 2 diabetes mellitus (T2DM) is rapidly becoming a global pandemic, currently affecting 8.8% of the world's population [1–3]. It causes significant suffering for those affected, increases mortality and costs the global economy $727 billion USD per year [1,2,4,5]. Additionally, diabetes is no longer a disease of wealthy nations. Approximately 79% of the 254 million people affected live in low- to middle-income countries (LMICs)[1,6]. The rising prevalence of T2DM in LMICs is particularly problematic since these countries have limited resources to implement effective interventions that are sustainable over time [2,7]and to treat the chronic comorbidities linked to T2DM[8].

LMICs display the fastest increase in the contribution of the global burden of diabetes [5]. Epidemiological studies show that development and urbanization in LMICs have led to a less active lifestyle [9,10] and an increased consumption of processed foods and sugary snacks with a high glycaemic index and [6,11]. Combined with an improvement in infectious disease management and population ageing [12], societal changes have expanded the proportional contribution of diabetes to the global burden of disease [6,13].Nutritional interventions can offer a highly cost-effective avenue to address T2DM [7].

Nutrition therapy, which promotes healthy eating patterns by targeting nutrient intake and/or portion size [14], can ameliorate glycaemic control, weight management, and delay complications of diabetes [3,15–17]. Good glycaemic regulation can also aid prevention of cardiovascular disease, which is the greatest cause of morbidity and mortality in T2DM [7,16]. These effects have also been observed in the absence of pharmacological agents, when people are unable to travel to healthcare facilities or when there is a lack of education and health literacy [3,7,11]. However, high quality research on T2DM prevention and management strategies has focused primarily on high-income countries [4]. Randomized controlled studies (RCTs), considered as a gold-standard for evidence-based health interventions [18], are less common in LMICs, with 78% of RCTs for non-communicable diseases recruiting participants in high-income countries [19].

Given the scarce number of trials available in many LMICs, development and implementation of interventions lacks rigorous scientific appraisal, or will often be based on the evidence gathered in HICs. Direct application of interventions tested in HICs may not be effective in LMICs, as they may not be sensitive to context-specific characteristics of communities in LMICs in terms of religion, socioeconomic status, composition of staple foods and cultural norms [8]. These factors strongly influence both prevalence and management of T2DM and hence the success of nutritional interventions [1,3,20]. In addition, assessment of outcomes following the termination of an intervention is fundamental to evaluate its effectiveness. Weight regain is frequent in people with T2DM, particularly when continuous support provided in weight loss interventions ends [21]. Measurement of glycated haemoglobin (HbA1c), the standard measure of glucose regulation, reflects the average blood glucose levels over the 2-3 months prior to measurement. Therefore, when evaluating the effects of an intervention on glucose control, it is essential that a 3-month follow-up assessment is in place. This is likely to be rare in LMICs, when critical analysis of evidence from high-income countries reveals that long-term follow-up is often limited to a maximum of 8-months post-intervention [17,22,23].

The purpose of this study is to systematically review RCTs carried out in LMICs assessing the short-term effectiveness of interventions focusing on nutrition therapy to improve glycaemic control in people with T2DM. Importantly, it will consider those interventions that have included a 3-month

For peer review only - http://bmjopen.bmj.com/site/about/guidelines.xhtml

For peer review only

post-intervention assessment of HbA1c to assess the primary outcome measure of potential benefits on glycaemic control following intervention completion. We expected that the present review would add to an earlier analysis of nutrition interventions for T2DM prevention in LMICs [24], thus providing an overview of high-quality research conducted to address the management of T2DM in LMICs.

METHODS

Search strategy and selection criteria

Following the steps laid out in our protocol (PROSPERO CRD42020188435), a systematic literature search was conducted to identify RCTs carried out in LMICs as defined by the Development Assistance Committee list of recipients of Official Development Assistance published in 2018[25]. Throughout this process the PRISMA Checklist was used to ensure proper reporting of the review[26]. The databases MEDLINE, EMBASE and Web of Science were searched, as well as Open Grey and the International Clinical Trials Registry Platform. The search was completed on the 3^{d} of July 2020. Search terms can broadly be divided into 4 categories: diabetes, nutrition, and diet, LMICs and RCTs (see Supplementary Material 1 for the Medline search). The references of relevant systematic reviews identified through literature searching were manually checked for further papers, as were the reference lists of included papers. Authors of unpublished clinical trials which were included based on protocols, were contacted by email to request results or manuscripts where available.

The inclusion and exclusion criteria followed the PICO framework [27]:

Types of studies:

- Published and unpublished randomized controlled trials that investigate nutritional therapy for T2DM.
- Minimum follow-up period of 12 weeks from the end of intervention.
- Full-text reports in any language.

Participants:

- Adults (≥18 years), male and female, any ethnicity.
- Residents of a low-, middle- or upper middle-income country as defined by the Development Assistance Committee list of recipients of Official Development Assistance for 2018-2020.
- Diagnosis of T2DM based on HbA1c ≥6.5%, random blood glucose test or oral glucose tolerance test ≥11.1mmol/l.
- Exclusion: prediabetes, metabolic syndrome without definitive T2DM diagnosis, type 1 diabetes, or gestational diabetes.

Interventions:

- Nutritional interventions delivered in person, in groups, through mobile/internet-based services or any other means.
- Food supplements/replacements
- Calorie/diet restrictions
- Lifestyle education interventions with a focus on diet.
- Exclusion: pharmacological interventions, preventative interventions

Comparators:

- No intervention/standard or minimal care/intervention that does not include nutritional therapy.

Outcomes:

For peer review only - http://bmjopen.bmj.com/site/about/guidelines.xhtml

- Primary: glycemic control (including HbA1c, fasting plasma glucose, insulin sensitivity/resistance)
- Secondary:
    - Symptoms e.g., reduction in polyuria, polydipsia, fatigue
    - Diabetic complications e.g., cardiovascular events, retinopathy, diabetic foot, nephropathy, neuropathy, hypo- and hyper-glycaemia
    - Psychological effects including quality of life and enjoyment of food.
    - Adverse effects including malnutrition & economic consequences of diet.

Quantitative outcomes only, either continuous or categorical

Screening was not limited by date or language and both published and unpublished work was reviewed. In LMICs, interventions must be able to be maintained once funding and services provided by the investigators have been withdrawn. In addition, HbA1c levels reflect average blood glucose levels over the 2-3 months prior to measurement. For this reason, studies were excluded if they had less than a 12-week follow-up period after the intervention had finished. Those studies where follow-up assessment was only carried out while the intervention was ongoing were excluded. Studies of a population with gestational or type 1 diabetes were also excluded as management strategies differ from those for T2DM [14,28]. Studies were included when the primary focus was nutritional therapy (*i.e.,* diet modification). The studies identified were imported into Rayyan [29] for screening by 2 independent researchers (LG and ST) after duplicates had been removed using EndNote Web. Conflicts between researchers were resolved by consensus, with the support of a third researcher when necessary (RP).

Data analysis

Based on the inclusion and exclusion criteria, 4 papers were included after full-text screening. Data were collected by LG and checked by ST using an adapted version of the Cochrane data collection form for RCTs and non-randomized studies [30]. Data on HbA1c and fasting plasma glucose were extracted. Where HbA1c and/or fasting plasma glucose levels were reported in different measuring units across papers, mean and standard deviation of results were converted to achieve a comparable set of results (*e.g.,* mmol/L to mg/dl; See Supplementary Material 2 for conversion). Other domains of extraction included study design characteristics, population, intervention, comparator, and secondary health outcomes. Outcomes extracted were the ones reporting follow-up data after completion of the intervention, which could be at 3 months or 6 months. The data extraction form included a template for Risk of Bias assessment based on version 2.0 of the Cochrane Risk-of-Bias instrument for randomized trials. Each domain was assessed to produce scores of “high”, “low”, or “some concern”. Data extraction was conducted independently and in duplicate.

Meta-analyses were conducted using Stata 16 (StataCorp LLC, College Station, TX) for the 2 primary outcomes (HbA1c and fasting blood glucose). Pooled mean differences were calculated with corresponding 95% confidence intervals. First, we pooled studies according to the time at which the follow-up assessment was undertaken (3 vs 6 months). Second, we generated an overall effect estimate.

Overall certainty of evidence was rated using the GRADE (Grading of Recommendations Assessment, Development and Evaluation; [31]) approach, where RCTs begin at the highest level of certainty and are downgraded if concerns arise in one or more of the following 5 domains: risk of bias,

For peer review only - http://bmjopen.bmj.com/site/about/guidelines.xhtml

inconsistency, indirectness, imprecision, and publication bias [32,33]. GRADE was completed independently by SS and RP, and consensus was reached with oversight from AW. Heterogeneity was reported using the I^2 statistic and the χ^2 test for homogeneity. Adhering to recommendations in the Cochrane Handbook, a heterogeneity score of 50% to 90% was considered as substantial, and a score of 75% to 100% was deemed evidence for considerable inconsistency [34]. As the number of eligible trials was inferior to 10, we did not proceed with using a funnel plot to evaluate publication bias, as originally planned, trials were scrutinized individually following recommendations by Guyatt and colleagues [31] to provide a final judgement of 'strongly suspected' or 'undetected' on the GRADEpro software tool.

Patient and Public Involvement

No patients were involved in this research study. Informal discussions with health professionals in Cameroon as part of another project raised the need to conduct the systematic review before co-designing a novel intervention with their patients.

RESULTS

The initial literature search yielded 5,075 results. After duplicates were removed, 1,948 records remained. Title and abstract screening of these records was carried out by 2 reviewers and gave a percentage agreement of 91% and a Cohen's kappa statistic of k=0.45 indicating moderate agreement [35]. Eighteen trial protocols were identified as potentially relevant for which authors were contacted for results. Of these, 14 did not provide a response, 2 had not yet completed analysis and 2 returned a published manuscript. After conflicting decisions were resolved, 44 studies remained for full text eligibility checking, and 4 studies met inclusion criteria (see Figure 1).

The 4 included papers (Supplementary Material 3) reported studies conducted in South Africa[36], Malaysia[37] and Iran[38,39], which are all upper-middle income countries (a subgroup of LMICs) according to the OECD [25]. Combined, the studies examined a sample size of 463 participants (mean study sample: 115.75, weighted standard deviation of sample size: 27.11). 234 participants had been assigned to the intervention arm, and 229 to the control arm. All studies recruited participants from medical or diabetic clinics; 2 were hospital based and 2 from community health centres.

All participants had been diagnosed with T2DM, had a weighted mean age of 57 years (SD: 6.4 years), and 63% (n=291) were female. 1 trial reported the presence of comorbidities (hypertension, dyslipidaemia, and a history of cardiovascular disease, 1 excluded those candidates with chronic conditions in addition to T2DM, and 2 trials did not provide information on comorbidity. The baseline weighted mean HbA1c of participants was 8.75% (SD: 1.38%) collected from the 4 trials, and the baseline weighted mean fasting blood glucose was of 154.73 mg/dl (SD: 5.41 mg/dl) collected from 3 studies.

Two trials [36,38] had delivered 8-week face-to-face group interventions, one had focused on group training sessions spread over 4 weeks (a total of 8 sessions)[39], and 1 evaluated a personalized online intervention delivered over 12 sessions [37]. All RCTs focused on nutritional education, but the interventions differed in their key features. 1 trial aimed to improve knowledge on the diabetes diet [36], 1 tailored the format of lessons to each individual's current Dietary Stage of Change (DSOC, a system to classify an individual's readiness to change a health-related behaviour)[37], 1 cantered

For peer review only - http://bmjopen.bmj.com/site/about/guidelines.xhtml

on the psychological factors known to influence dietary habits in diabetes[38], and the last 1 combined general diabetes nutrition education with a jogging regime[39]. Shared themes to all 3 interventions were portion control and healthy food choices.

The common outcome used to assess glycaemic control was HbA_{1c}, and 3 studies additionally measured fasting blood glucose. Assessment of glycaemic control took place at baseline across all studies and 1 study at end of intervention. 2 studies reported follow-up data on glycaemic control at 3-months after intervention completion, and 2 at 6-months. 1 study had an additional follow-up assessment at 12 months[36]. Dropouts were present across all interventions. Overall, both HbA1c and fasting blood glucose were found to have unclear risk of bias in 1 study and evidence for high risk of bias in a second 1 (Figure 2). Low risk of bias was deemed in the measurement of the outcomes due to the standardized procedure adopted to measure glycaemic control, but 2 studies lacked sufficient details on the protocol of for participant allocation and a lack of a-priori protocol prevented the assessment of selective reporting bias. In 1 study[39], participants were excluded if they missed 2 sessions of training, but there was no indication on the numbers of participants who were therefore also withdrawn from the trial. A 'high risk' judgment was made on the domain complete accounting of patients.

Table 1 provides a summary of findings. Figures 3 and 4 present the pooled analysis for HbA1c and fasting blood glucose. As 2 studies measured these outcomes at a post-intervention follow-up of 3 months and 2 6 months, pooled analyses were split by timepoint of assessment. At 3 months, the nutritional interventions were more effective than standard protocol, with the pooled mean differences at –1.11% (95% CI –1.64 to –0.59) for hbA1c and –23.57 mg/dl (95% CI –44.3 to –2.84) for fasting blood glucose. The advantage was no longer observed in studies with a 6-month follow-up assessment, with pooled mean differences at -0.09% (95% CI -1.10 to 0.91) for hba1c and 9mg/dl (95% CI -7.55 to 25.55) for fasting blood glucose. Overall, nutritional therapy was not found to have a meaningful impact on Hba1c (mean difference: -0.63%, 95% CI -1.46 to 0.21; 4 studies, n=463, GRADE=very low) nor fasting blood glucose (mean difference: -0.63%, 95% CI -1.46 to 0.21; 3 studies, n=381, GRADE=very low) in the short-term after completion of the intervention. Note, hbA1c provides an average measure of blood glucose over the previous 3 months, due to the lifecycle of red blood cells. Further, the effects of nutritional interventions are likely to be sustained in the early months post-intervention. We would therefore not expect the difference in timepoint assessment to have a clinically meaningful impact on the reported effects. However, the small number of studies that met inclusion criteria does not permit definite conclusions to be reached.

For peer review only - http://bmjopen.bmj.com/site/about/guidelines.xhtml

Table 1. Summary of findings for primary outcomes.

Outcomes	Anticipated absolute effects* (95% CI)		Relative effect (95% CI)	№ of participants (studies)	Certainty of the evidence (GRADE)
	**Risk with standard care**	**Risk with Nutritional therapy**			
HbA1c<br>Scale from: 0% to 100%<br>follow up: range 3 months to 6 months	The mean HbA1c ranged from **8.13-10.3%**	MD **0.63 % lower**<br>(1.47 lower to 0.21 higher)	-	463<br>(4 RCTs)	⨁◯◯◯<br>VERY LOW [a,b,c,d]
Fasting Blood Glucose<br>follow up: range 3 months to 6 months	The mean fasting blood glucose ranged from **136.8 to 153.64 mg/dl.**	MD **13.63 mg/dl lower**<br>(37.61 lower to 10.34 higher)	-	381<br>(3 RCTs)	⨁◯◯◯<br>VERY LOW [a,d,e,f]

***The risk in the intervention group** (and its 95% confidence interval) is based on the assumed risk in the comparison group and the **relative effect** of the intervention (and its 95% CI).

CI: Confidence interval; **MD:** Mean difference

GRADE Working Group grades of evidence
High certainty: We are very confident that the true effect lies close to that of the estimate of the effect
Moderate certainty: We are moderately confident in the effect estimate: The true effect is likely to be close to the estimate of the effect, but there is a possibility that it is substantially different
Low certainty: Our confidence in the effect estimate is limited: The true effect may be substantially different from the estimate of the effect
Very low certainty: We have very little confidence in the effect estimate: The true effect is likely to be substantially different from the estimate of effect

For peer review only - http://bmjopen.bmj.com/site/about/guidelines.xhtml

Table 1. Summary of findings for primary outcomes.

Outcomes	**Anticipated absolute effects*** (95% CI)		Relative effect (95% CI)	№ of participants (studies)	Certainty of the evidence (GRADE)
	**Risk with standard care**	**Risk with Nutritional therapy**			

Explanations

a. Rated down for the small number of trials. 2 trials additionally presented unclear or high risk in several domains: randomization process, missing outcome data, and selection of the reported result.

b. Heterogeneity: p = 0.01. Downgrading carried out because p<0.01 is considered as the threshold for consistency[40]. Small sample sizes, with an I2 of 76%, reflecting considerable inconsistency.

c. The optimal information size (OIS) is 106 (based on a power of 0.80 being considered as adequate), and the total sample size of the analysis is above this (n=463). However, rated down because the confidence intervals of 2 trials contain the null effect, and though there is no harm from the intervention, clinical judgement suggests that an absolute effect size of 0.63 is not sufficiently high to judge in favor of the nutritional intervention.

d. Rated down because of the small sample size of the trials, the inability to access some full text manuscripts that might have met the inclusion criteria, and likely existence of ongoing clinical trials that may have been missed.

e. Heterogeneity: p = 0.000. Downgrading carried out because p<0.01 is considered as the threshold for consistency [40]. Small sample sizes, with an I2 of 92%, reflecting considerable inconsistency.

f. The optimal information size (OIS) is 730, the total sample size of included trials is below this value (n=381). Further the confidence intervals do not exclude no effect (-7.55 to 25.55).

For peer review only - http://bmjopen.bmj.com/site/about/guidelines.xhtml

Secondary outcomes are presented in Supplementary Material 4. Briefly, 1 study reported [38] a notable improvement in perceived adherence to a healthy diet at 3-months after the intervention, whereas another study reported the intervention to generate benefits for dietary knowledge, attitudes and behaviour at 6 months [37]. Two studies further examined the impact of nutrition education on lipid profile and on body mass index (BMI), biomarkers of elevated risk of diabetes [41]. HDL lipoprotein-cholesterol appears to improve following nutritional therapy when the follow-up assessment occurs at 3 months [39], but this is no longer so when the follow-up occurs at 6-months after intervention end [36]. At follow-up, nutrition therapy does not seem to impact other markers of increased risk of diabetes complications (*e.g.,* heart disease) evaluated by the trials. However, the quality of the evidence is overall very poor (Grade = Very Low). No further information was reported on adverse events nor other pre-specified secondary outcomes.

Apart from grouping analyses according to timepoint of follow-up assessment, no further subgroup analyses were conducted given the limited number of studies eligible for inclusion. Table 1 presents GRADE judgements. The certainty of evidence on the short-term benefits of nutrition therapy for treating type 2 diabetes mellitus in low- to middle- income countries is overall very low. This is primarily due to the small number of RCTs with a follow-up conducted in these countries, the inconsistency between studies, high level of imprecisions and likelihood of publication bias.

Supplementary Material 5 presents the completed PRISMA checklist.

CONCLUSION

This systematic review set out to synthesize the evidence on the short-term effectiveness of nutritional therapy for glycaemic control in people living with T2DM in LMICs. Only RCTs with a follow-up of 3-months after intervention completion were included given that HbA1c, the gold-standard for assessment of glucose regulation in T2DM, provides an aggregate measure of the previous 3 months. Only 4 trials met the eligibility criteria, where the main component of the intervention was nutrition education, delivered via a mobile device or face-to-face group training. Overall, the small number of trials, considerable heterogeneity, and concerns over the methodology and reporting in some trials indicate that evidence available from RCTs at present is of very low quality.

Grounded on very low certainty of the evidence, the results of RCTs suggest that nutrition education does not yield significant benefits for HbA1c levels or fasting blood glucose in people with T2DM living in LMICs. It may have some benefits for perceived adherence to diet and dietary knowledge, attitudes, and behaviour, but not other markers for diabetic complications, such as LDL lipoprotein-cholesterol, triglycerides, BMI, systolic and diastolic blood pressure. Findings from this review therefore contrast optimistic results from community-based prevention strategies and lifestyle interventions, which have shown to reduce the risk of diabetes[24,42] and improve glycaemic control[4,7,42]. In line with recommendations generated from research in HIC [43], the discrepancy in findings indicates that diabetes management may require a multi-faceted approach, with equal importance given to nutrition, physical activity, and diabetes education and medication administered when necessary. Within the context of the low-resourced healthcare systems found in LMICs, it may be tempting to seek cost-saving solutions. However, this review suggests that reducing diabetes management to short, nutrition education programs may not be sufficient to address the growing burden of diabetes in LMICs, particularly given that the potential impact on glycaemic results appears to dissipate after 6 months following intervention completion.

For peer review only - http://bmjopen.bmj.com/site/about/guidelines.xhtml

The small number of RCTs identified in this review additionally reflects consensus that there is a general absence of large experimental research around diabetes management interventions in LMICs[4,44]. While conventional RCTs are considered the gold-standard for evidence-based medicine[18], which are crucial to ensure maximization of available healthcare resources[45], the use of pragmatic trials may be more appropriate to derive evidence in LMICs[46]. Short-term interventions funded by overseas agencies rarely involve local stakeholders and the research findings are less likely to be made available locally, although they may gain more international attention when undertaken by an agency from an HIC[47]. As demonstrated by this review, adopting an RCT design may also not add value to available evidence if it is not of high standard or lacks longer term assessment. It is in countries where follow-up is least reported that continued assessment is most critical, as sustainability is essential if it is to benefit those living in the community under investigation, and if research agendas are to be set according to local needs[48].

The trials identified in this review benefit from being carried out in 3 separate countries (Malaysia, South Africa and Iran) which vary greatly in terms of culture and diet. However, all 3 countries where the trials were run are classified in the upper-middle income country subgroup of LMICs according to DAC[25], which reflects other studies showing these countries to produce the most NCD-related literature[19]. This may be due to the high prevalence of diabetes in these countries as they progress through the epidemiological transition most rapidly and hence have more relevant literature[49]. However, it may also be attributable to the inequity in availability and access to medical treatment that is widespread in low-income countries, which remains a problem even as non-communicable disease research increases in LMICs as a whole[1,19,50]. Though funding for nutrition research is increasing for these countries, the majority continues to be directed towards addressing undernutrition [51].

By using a robust evidence synthesis method, including Cochrane's Risk of Bias instrument 2.0 and the GRADE system, this review demonstrates that drawing conclusions from RCTs conducted in these countries may be premature if the quality of the evidence is not carefully examined. Given the short-term nature of the interventions assessed in eligible trials (8 weeks), measuring HbA1c levels at follow-up provides a more accurate indication of the impact of these types of interventions on glycaemic regulation. However, the focus on RCTs that included a follow-up signifies that this review did not capture other types of interventions. Further, the definition of nutritional therapy lacks standardization. Here, we based our definition on the American Diabetes Association regarding its content[14], but note that none of the eligible trials had interventions where nutrient or whole-food intake was directly manipulated, as seen in HIC (e.g., DIRECT trial[52]) nor where they are delivered by a registered dietitian, as recommended by ADA. Growing evidence points to the important role of community health workers for health promotion in LMICs[7,24] and to adapt research designs to setting in which they are implemented[53]. Hence, being too restrictive in eligibility criteria when synthesizing the evidence may hinder advances in evidence-guided improvements in care.

In conclusion, very low certainty of evidence suggests that nutritional therapy alone may not be sufficient to improve glycaemic control in people living with T2DM in LMICs, when control is assessed even a few months after completion of the intervention. It may, however, have some benefits for perceived adherence to diet and dietary knowledge and behaviours. As T2DM becomes a growing problem in LMICs, greater efforts are needed to build capacity for high-quality, context-appropriate, and long-term research, particularly in lowest-income countries. Improved quality of

For peer review only - http://bmjopen.bmj.com/site/about/guidelines.xhtml

evidence will better guide decision-makers and health professionals to implement interventions that are financially sustainable and effective to reduce individual and societal burden of T2DM in LMICs.

ETHICS STATEMENT

Not applicable/No human participants included

CONTRIBUTORSHIP STATEMENT

SS and EG conceptualized the idea and led the review process. EG generated the first draft of the manuscript, and SS further refined it to adhere to the latest guidance on evaluation of the quality of evidence (*i.e.,* GRADE). RP led the design of the review methodology. AW guided the statistical aspects of the review and was responsible for the standardization of the data. Both RP and AW edited the manuscript in accordance with their expertise. All authors approved the final version of the manuscript and agree to be accountable for all aspects of the work.

COMPETING INTERESTS

There are no competing interests. The outcome of this systematic review constitutes part of the preliminary research conducted to inform the development and assessment of interventions in Cameroon.

FUNDING

SS, RP, and AW are supported by the NIHR Biomedical Research Centre at University Hospitals of Bristol and Weston NHS Foundation Trust and the University of Bristol. The views expressed are those of the authors and not necessarily those of the NIHR or the Department of Health and Social Care.

DATA SHARING

No additional data is available beyond the information provided by articles included. Data regarding the systematic review process can be requested from the corresponding author.

REFERENCES

1 International Diabetes Federation. *IDF Diabetes Atlas, 9th edition*. Brussels: 2019.

2 Glezeva N, Chisale M, McDonald K, *et al.* Diabetes and complications of the heart in Sub-Saharan Africa: An urgent need for improved awareness, diagnostics and management. Diabetes Res. Clin. Pract. 2018;137:10–9. doi:10.1016/j.diabres.2017.12.019

3 Mechanick JI, Marchetti AE, Apovian C, *et al.* Diabetes-specific nutrition algorithm: A transcultural program to optimize diabetes and prediabetes care. *Curr Diab Rep* 2012;12:180–94. doi:10.1007/s11892-012-0253-z

4 Afable A, Karingula NS. Evidence based review of type 2 diabetes prevention and management in low and middle income countries. *World J Diabetes* 2016;7:209. doi:10.4239/wjd.v7.i10.209

5 World Health Organisation. Global report on diabetes. 2016.https://www.who.int/publications/i/item/9789241565257 (accessed 24 Apr 2020).

6 Mattei J, Malik V, Wedick NM, *et al.* Reducing the global burden of type 2 diabetes by

For peer review only - http://bmjopen.bmj.com/site/about/guidelines.xhtml

improving the quality of staple foods: The Global Nutrition and Epidemiologic Transition Initiative. *Global Health* 2015;11. doi:10.1186/s12992-015-0109-9

7 Alaofè H, Asaolu I, Ehiri J, *et al.* Community Health Workers in Diabetes Prevention and Management in Developing Countries. Ann. Glob. Heal. 2017;83:661–75. doi:10.1016/j.aogh.2017.10.009

8 Checkley W, Ghannem H, Irazola V, *et al.* Management of NCD in low- And middle-income countries. Glob. Heart. 2014;9:431–43. doi:10.1016/j.gheart.2014.11.003

9 Ley SH, Hamdy O, Mohan V, *et al.* Prevention and management of type 2 diabetes: Dietary components and nutritional strategies. Lancet. 2014;383:1999–2007. doi:10.1016/S0140-6736(14)60613-9

10 Ng SW, Popkin BM. Time use and physical activity: A shift away from movement across the globe. *Obes Rev* 2012;13:659–80. doi:10.1111/j.1467-789X.2011.00982.x

11 Micikas M, Foster J, Weis A, *et al.* A Community Health Worker Intervention for Diabetes Self-Management Among the Tz'utujil Maya of Guatemala. *Health Promot Pract* 2015;16:601–8. doi:10.1177/1524839914557033

12 Weisman A, Fazli GS, Johns A, *et al.* Evolving Trends in the Epidemiology, Risk Factors, and Prevention of Type 2 Diabetes: A Review. Can. J. Cardiol. 2018;34:552–64. doi:10.1016/j.cjca.2018.03.002

13 Boutayeb A. The double burden of communicable and non-communicable diseases in developing countries. Trans. R. Soc. Trop. Med. Hyg. 2006;100:191–9. doi:10.1016/j.trstmh.2005.07.021

14 Evert AB, Dennison M, Gardner CD, *et al.* Nutrition therapy for adults with diabetes or prediabetes: A consensus report. Diabetes Care. 2019;42:731–54. doi:10.2337/dci19-0014

15 Perry HB, Zulliger R, Rogers MM. Community health workers in low-, middle-, and high-income countries: An overview of their history, recent evolution, and current effectiveness. In: *Annual Review of Public Health*. Annual Reviews Inc. 2014. 399–421. doi:10.1146/annurev-publhealth-032013-182354

16 Agee MD, Gates Z, Irwin PM. Effect of medical nutrition therapy for patients with type 2 diabetes in a low-/no-cost clinic: A propensity score-matched cohort study. *Diabetes Spectr* 2018;31:83–9. doi:10.2337/ds16-0077

17 Taylor R. Calorie restriction for long-term remission of type 2 diabetes. Clin. Med. J. R. Coll. Physicians London. 2019;19:37–42. doi:10.7861/clinmedicine.19-1-37

18 Murad MH, Asi N, Alsawas M, *et al.* New evidence pyramid. Evid. Based. Med. 2016;21:125–7. doi:10.1136/ebmed-2016-110401

19 Fan H, Song F. An assessment of randomized controlled trials (RCTs) for non-communicable diseases (NCDs): More and higher quality research is required in less developed countries. *Sci Rep* 2015;5. doi:10.1038/srep13221

20 Forouhi NG, Misra A, Mohan V, *et al.* Dietary and nutritional approaches for prevention and management of type 2 diabetes. *BMJ* 2018;361. doi:10.1136/bmj.k2234

21 Guare JC, Wing RR, Grant A. Comparison of Obese NIDDM and Nondiabetic Women: Short- and Long-Term Weight Loss. *Obes Res* 1995;3:329–35. doi:10.1002/j.1550-8528.1995.tb00158.x

22 Steven S, Hollingsworth KG, Al-Mrabeh A, *et al.* Very low-calorie diet and 6 months of weight stability in type 2 diabetes: Pathophysiological changes in responders and nonresponders.

For peer review only

For peer review only - http://bmjopen.bmj.com/site/about/guidelines.xhtml

Diabetes Care 2016;39:808–15. doi:10.2337/dc15-1942

23 Pastors JG, Warshaw H, Daly A, *et al.* The evidence for the effectiveness of medical nutrition therapy in diabetes management. Diabetes Care. 2002;25:608–13. doi:10.2337/diacare.25.3.608

24 Shirinzadeh M, Afshin-Pour B, Angeles R, *et al.* The effect of community-based programs on diabetes prevention in low- and middle-income countries: A systematic review and meta-analysis. Global. Health. 2019;15. doi:10.1186/s12992-019-0451-4

25 Organisation for Economic Co-operation and Development. Development Assistance Committee List of Official Development Assistance Recipients: Effective for reporting on 2018, 2019 and 2020 flows. 2018. http://www.oecd.org/dac/financing-sustainable-development/development-finance-standards/DAC_List_ODA_Recipients2018to2020_flows_En.pdf: OECD (accessed 24 May 2021).

26 Moher D, Liberati A, Tetzlaff J, *et al.* Preferred reporting items for systematic reviews and meta-analyses: The PRISMA statement. PLoS Med. 2009;6:e1000097. doi:10.1371/journal.pmed.1000097

27 Richardson W, Wilson M, Nishikawa J, *et al.* The well-built clinical question: a key to evidence-based decisions - PubMed. *ACP J Club* 1995;123:A12–3.https://pubmed.ncbi.nlm.nih.gov/7582737/ (accessed 5 May 2021).

28 14. Management of diabetes in pregnancy: Standards of medical care in diabetesd2019. *Diabetes Care* 2019;42:S165–72. doi:10.2337/dc19-S014

29 Ouzzani M, Hammady H, Fedorowicz Z, *et al.* Rayyan-a web and mobile app for systematic reviews. *Syst Rev* 2016;5:210. doi:10.1186/s13643-016-0384-4

30 Higgins J, Green S. *Cochrane Handbook for Systematic Reviews of Interventions.* Version 5. 2011.

31 Guyatt G, Oxman AD, Akl EA, *et al.* GRADE guidelines: 1. Introduction - GRADE evidence profiles and summary of findings tables. *J Clin Epidemiol* 2011;64:383–94. doi:10.1016/j.jclinepi.2010.04.026

32 Guyatt GH, Oxman AD, Vist GE, *et al.* GRADE: An emerging consensus on rating quality of evidence and strength of recommendations. *BMJ* 2008;336:924–6. doi:10.1136/bmj.39489.470347.ad

33 Schünemann H, Brożek J, Guyatt G, *et al.* Handbook for Grading the Quality of Evidence And the Strength of Recommendation Using the GRADE Approach. 2013.https://gdt.gradepro.org/app/%0Ahandbook/handbook.html. (accessed 25 Oct 2020).

34 Deeks J, Higgins J, Altman D. Analyzing data and undertaking meta-analyses. In: Higgins J, S G, eds. *Cochrane handbook for systematic reviews of interventions version 5.0.0*. Chichester: : Wiley 2008.

35 Landis JR, Koch GG. The Measurement of Observer Agreement for Categorical Data. *Biometrics* 1977;33:159. doi:10.2307/2529310

36 Muchiri JW, Gericke GJ, Rheeder P. Effect of a nutrition education programme on clinical status and dietary behaviours of adults with type 2 diabetes in a resource-limited setting in South Africa: A randomised controlled trial. *Public Health Nutr* 2016;19:142–55. doi:10.1017/S1368980015000956

37 Ramadas A, Chan CKY, Oldenburg B, *et al.* Randomised-controlled trial of a web-based dietary intervention for patients with type 2 diabetes: Changes in health cognitions and glycemic

For peer review only

control. *BMC Public Health* 2018;18:1–13. doi:10.1186/s12889-018-5640-1

38 Salahshouri A, Zamani Alavijeh F, Mahaki B, *et al.* Effectiveness of educational intervention based on psychological factors on achieving health outcomes in patients with type 2 diabetes. *Diabetol Metab Syndr* 2018;10:67. doi:10.1186/s13098-018-0368-8

39 Askari A, Jeihooni A, Kashfi S, *et al.* The Effect of Educational Program Based on Belief, Attitude, Subjective Norm, and Enabling Factors Model on Changing the Metabolic Indices in Elderly Patients with Type II Diabetes. *Int J Prev Med* 2018;9:74.

40 Guyatt GH, Oxman AD, Kunz R, *et al.* GRADE guidelines: 7. Rating the quality of evidence - Inconsistency. *J Clin Epidemiol* 2011;64:1294–302. doi:10.1016/j.jclinepi.2011.03.017

41 Gray N, Picone G, Sloan F, *et al.* Relation between BMI and diabetes mellitus and its complications among US older adults. *South Med J* 2015;108:29–36. doi:10.14423/SMJ.0000000000000214

42 Gyawali B, Bloch J, Vaidya A, *et al.* Community-based interventions for prevention of Type 2 diabetes in low-and middle-income countries: a systematic review. *Health Promot Int* 2019;34:1218–30. doi:10.1093/heapro/day081

43 Association AD. 5. Facilitating behavior change and well-being to improve health outcomes: Standards of medical care in diabetes−2021. *Diabetes Care* 2021;44:S53–72. doi:10.2337/dc21-S005

44 Alemayehu C, Mitchell G, Nikles J. Barriers for conducting clinical trials in developing countries- a systematic review. Int. J. Equity Health. 2018;17. doi:10.1186/s12939-018-0748-6

45 Siddiqi K, Newell JN. Editorials Putting evidence into practice in low-resource settings. 2005. http://www. (accessed 12 May 2021).

46 English M, Karumbi J, Maina M, *et al.* The need for pragmatic clinical trials in low and middle income settings - taking essential neonatal interventions delivered as part of inpatient care as an illustrative example. *BMC Med* 2016;14:1–7. doi:10.1186/s12916-016-0556-z

47 Franzen SRP, Chandler C, Siribaddana S, *et al.* Strategies for developing sustainable health research capacity in low and middle-income countries: A prospective, qualitative study investigating the barriers and enablers to locally led clinical trial conduct in Ethiopia, Cameroon and Sri Lanka. *BMJ Open* 2017;7. doi:10.1136/bmjopen-2017-017246

48 Laabes EP, Desai R, Zawedde SM, *et al.* How much longer will Africa have to depend on western nations for support of its capacity-building efforts for biomedical research? *Trop Med Int Heal* 2011;16:258–62. doi:10.1111/j.1365-3156.2010.02709.x

49 Defo BK. Demographic, epidemiological, and health transitions: Are they relevant to population health patterns in Africa? *Glob Health Action* 2014;7. doi:10.3402/gha.v7.22443

50 Chow CK, Ramasundarahettige C, Hu W, *et al.* Availability and affordability of essential medicines for diabetes across high-income, middle-income, and low-income countries: a prospective epidemiological study. *Lancet Diabetes Endocrinol* 2018;6:798–808. doi:10.1016/S2213-8587(18)30233-X

51 Perez-Escamilla R, Bermudez O, Buccini GS, *et al.* Nutrition disparities and the global burden of malnutrition. *BMJ* 2018;361. doi:10.1136/bmj.k2252

52 Lean ME, Leslie WS, Barnes AC, *et al.* Primary care-led weight management for remission of type 2 diabetes (DiRECT): an open-label, cluster-randomised trial. *Lancet* 2018;391:541–51. doi:10.1016/S0140-6736(17)33102-1

53 Bekele H, Asefa A, Getachew B, *et al.* Barriers and Strategies to Lifestyle and Dietary Pattern

For peer review only - http://bmjopen.bmj.com/site/about/guidelines.xhtml

Interventions for Prevention and Management of TYPE-2 Diabetes in Africa, Systematic Review. J. Diabetes Res. 2020; 7948712. doi:10.1155/2020/7948712

ACKNOWLEDGEMENTS

We thank Prof. Simeon Pierre Choukem (University of Dschang, Dschang, Cameroon) for his insight on nutritional interventions in Cameroon for patients of type 2 diabetes mellitus, and for encouraging us to pursue this project.

FIGURE LEGENDS

Figure 1: PRISMA study flow.

Figure 2. Risk of bias of individual trials, by primary outcome measure.

Figure 3. Change in HbA_{1c} at follow-up assessment, split according to timepoint of assessment (3- or 6-months) and overall.

Figure 4. Change in fasting blood glucose at follow-up assessment, split according to timepoint of assessment (3- or 6-months) and overall.

For peer review only

For peer review only - http://bmjopen.bmj.com/site/about/guidelines.xhtml

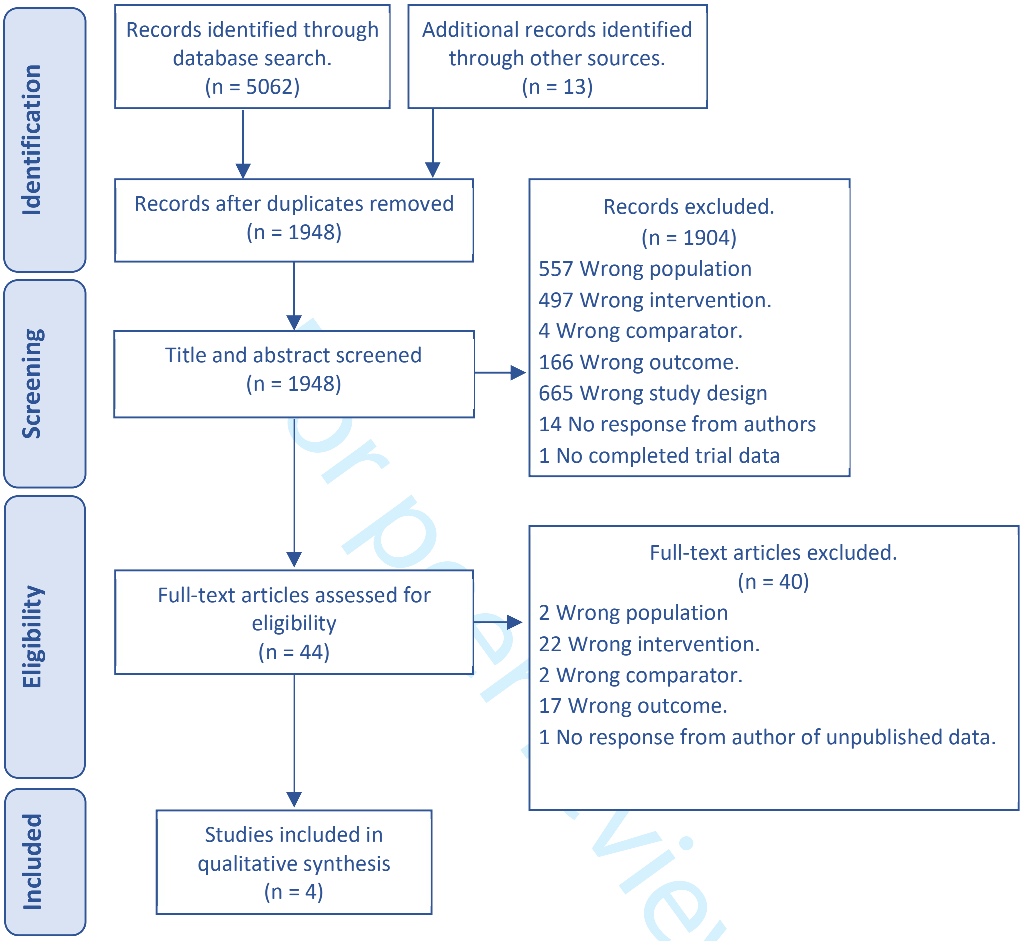
Identification
Screening
Eligibility
Included
Records identified through database search. (n = 5062)
Additional records identified through other sources. (n = 13)
Records after duplicates removed (n = 1948)
Title and abstract screened (n = 1948)
Records excluded. (n = 1904)
557 Wrong population
497 Wrong intervention.
4 Wrong comparator.
166 Wrong outcome.
665 Wrong study design
14 No response from authors
1 No completed trial data
Full-text articles assessed for eligibility (n = 44)
Full-text articles excluded. (n = 40)
2 Wrong population
22 Wrong intervention.
2 Wrong comparator.
17 Wrong outcome.
1 No response from author of unpublished data.
Studies included in qualitative synthesis (n = 4)

Outcome: HbA1c	Randomization process	Deviations from intended interventions	Missing outcome data	Measurement of the outcome	Selection of the reported result	Overall bias
Askari (2018)[39]	Some concerns	Some concerns	High risk	Low risk	Some concerns	High Risk
Muchiri (2016)[36]	Low risk	Low risk	Low risk	Low risk	Low risk	Low Risk
Ramadas (2018)[37]	Low risk	Low risk	Low risk	Low risk	Low risk	Low Risk
Salahshouri (2018)[38]	Some concerns	Low risk	Some concerns	Low risk	Some concerns	Some concerns

Outcome: Fasting blood glucose	Randomization	Deviations from intended interventions	Missing outcome data	Measurement of the outcome	Selection of the reported result	Overall bias
Askari (2018)[39]	Some concerns	Low risk	High risk	Low risk	Some concerns	High Risk
Muchiri (2016)[36]	N/A	N/A	N/A	N/A	N/A	N/A
Ramadas (2018)[37]	Low risk	Low risk	Low risk	Low risk	Low risk	Low Risk
Salahshouri (2018)[38]	Some concerns	Low risk	Some concerns	Low risk	Some concerns	Some concerns

For peer review only

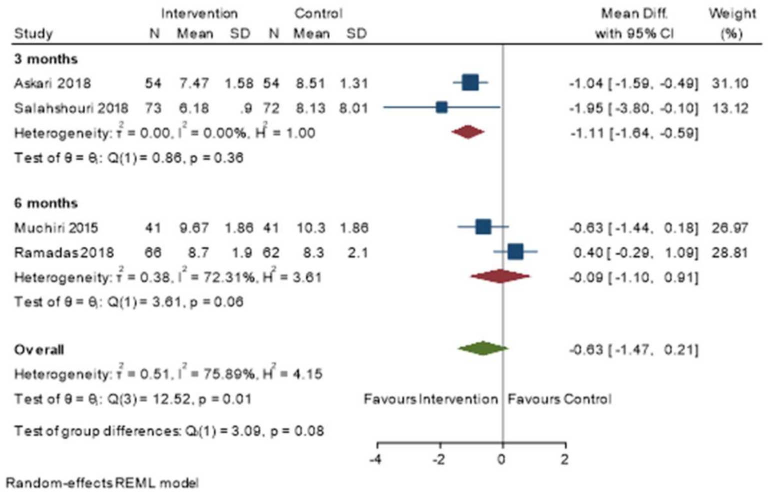

For peer review only

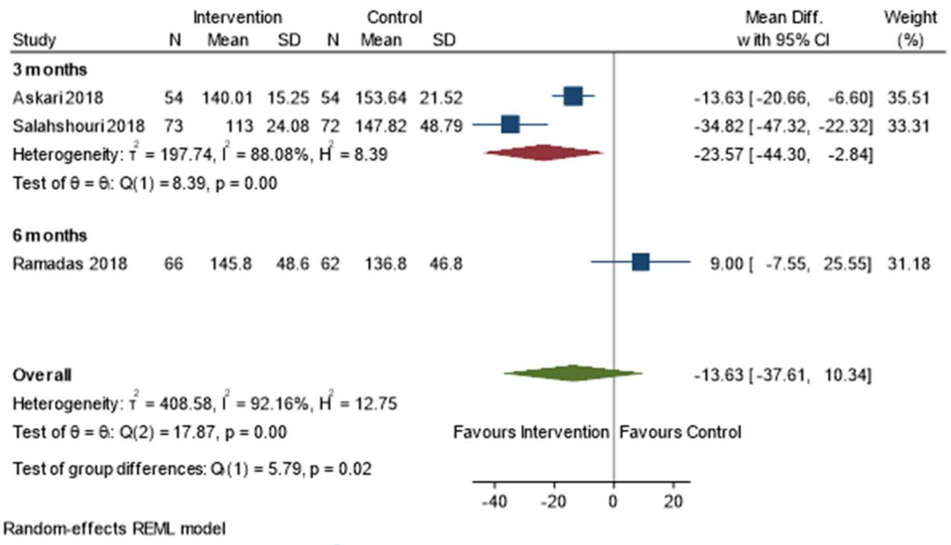

peer review only

SUPPLEMENTARY MATERIAL 1

Title: Short-term effectiveness of nutritional therapy to treat type 2 diabetes in low- and middle-income countries: systematic review of randomized-controlled trials.

*Elizabeth Guilbert[1,2], Rachel Perry[3], Alex Whitmarsh[3], Sarah Sauchelli[3]**

MEDLINE search strategy and number of results at each stage

1	diabetes mellitus/ or exp diabetes mellitus, type 2/ or exp hyperglycemia/ or exp metabolic syndrome/ (273807)
2	(diabet* adj5 treat*).tw. (64270)
3	(diabet* adj5 diagnos*).tw. (24777)
4	1 or 2 or 3 (322372)
5	exp Diet/ (261138)
6	*nutrition therapy/ or exp diet therapy/ (52679)
7	exp Nutrition Policy/ (9911)
8	exp nutrition assessment/ (13903)
9	nutrition disorders/ or exp overnutrition/ (213134)
10	((health* or wellness or weight or diet*) adj2 (treat* or Interven* or change* or improve*)).tw. (147274)
11	exp Food Labeling/ (3392)
12	5 or 6 or 7 or 8 or 9 or 10 or 11 (576981)
13	(afghan* or angola* or bangladesh* or benin* or bhutan* or "burkina fasso" or burundi* or cambodia* or "central african republic" or chad* or comoros or congo* or "democratic republic of the congo" or Djibouti* or eritrea* or ethiopia* or gambia* or guinea* or guinea-bissau or haiti* or kiribati* or "lao people's democratic republic" or lao or lesotho* or liberia* or madagasca* or malawi* or mali* or mauritania* or mozambique or myanmar or nepal* or niger or rwanda* or "sao tome and principe" or senegal* or "sierra leone" or "solomon islands" or somalia* or "south sudan" or sudan* or tanzania* or "timor-leste" or togo or tuvalu* or uganda* or vanuatu or yemen or zambia*).tw. (792163)
14	(armenia* or bolivia* or "cabo verde" or "cape verde" or cameroon* or congo* or "cote d'ivoire" or egypt* or "el salvador" or eswatini or georgia* or ghana* or guatemala* or honduras or india* or indonesia* or jordan* or kenya* or kosov* or kyrgyzstan or micronesia* or moldova* or mogolia* or morocc* or nicaragua* or nigeria* or pakistan* or "papua new guinea" or philippin* or "sri lanka" or "syrian arab republic" or syria* or tajikistan or tokelau or tunisia* or Ukrain* or uzbekistan or "viet nam" or "west bank and gaza strip" or "democratic people's republic of korea" or zimbabwe*).tw. (345506)
15	(albania* or algeria* or "antigua and barbuda" or argentin* or azerbaijan or belarus or belize or "bosnia and herzegovina" or botswana* or brazil* or china or colombia* or "cook islands" or "costa rica" or cuba* or dominica* or "dominican republic" or ecuador* or "equatorial guinea" or fiji or "former yugoslav republic of macedonia" or gabon or grenada or guyana* or iran* or iraq* or jamaica* or kazakhstan or lebanon or libya or Malaysia or Maldives or "marshall islands" or Mauritius or Mexico or Montenegro or Montserrat or namibia* or nauru or niue or palau or panama or paraguay or peru* or "saint helena" or "saint lucia" or "saint vincent and the grenadines" or samoa* or serbia* or "south africa" or suriname or thailand or tonga* or turkey or turkmenistan or venezuela* or "wallis and futuna").tw. (507391)
16	developing country.mp. or Developing Countries/ (75649)

17	((developing or less* developed or under developed or underdeveloped or middle income or low* income or deprived or poor) adj (countr* or nation or population)).tw. (75342)
18	13 or 14 or 15 or 16 or 17 (1632917)
19	randomi* controlled trial.tw. (81488)
20	controlled clinical trial.pt. (93000)
21	randomized controlled trial.pt. (479114)
22	random*.ab. (988614)
23	trial.ab. (448719)
24	19 or 20 or 21 or 22 or 23 (1388842)
25	4 and 12 and 18 and 24 (630)

For peer review only

SUPPLEMENTARY MATERIAL 2

Title: Short-term effectiveness of nutritional therapy to treat type 2 diabetes in low- and middle-income countries: systematic review of randomized-controlled trials.

*Elizabeth Guilbert[1,2], Rachel Perry[3], Alex Whitmarsh[3], Sarah Sauchelli[3]**

Where included studies differed in measuring units used for glycated hemoglobin (HbA1c), fasting blood glucose, and other biomarkers these were converted to enable comparison. The following formulas were applied:

Fasting Blood Glucose (Table 1): Three studies reported fasting blood glucose in milligrams per deciliter (mg/dL), whereas one study[1] measured fasting blood glucose in millimoles per liter (mmol/L). The formula applied for conversion was: mg/dL = 18.01*mmol/l [2,3].

	**Baseline M(SD)**		**Follow-up (6 months post-intervention) M(SD)**	
	**Original (mmol/L)**	**Converted (mg/dl)**	**Original (mmol/L)**	**Converted (mg/dl)**
**Experimental Intervention**	8.0 (3.9)	160.2 (70.2)	8.1 (2.7)	145.8 (48.6)
**Control intervention**	8.3 (2.9)	149.4 (52.2)	7.8 (2.6)	136.8 (46.8)

Table 1: Unit transformation for Fasting Blood Glucose (Ramadas[1]). M: Mean, SD: Standard Deviation

HDL and LDL lipoprotein-cholesterol (Tables 2 and 3): The two studies that included biomarkers of diabetes complications used different units. Therefore, values in mmol/l were converted to mg/dl using the following formula: mg/dL = 38.67*mmol/l [4]

	**Baseline M(SD)**		**Follow-up (6 months post-intervention) M(SE)**	
	**Original (mmol/L)**	**Converted (mg/dl)**	**Original (mmol/L)**	**Converted (mg/dl)**
**Experimental Intervention**	3.00 (0.90)	116.67 (34.80)	2.66 (0.08)	103.74(3.12)
**Control intervention**	3.10(0.90)	119.88 (34.80)	2.74 (0.08)	106.86 (3.12)

Table 2: Unit transformation for LDL lipoprotein-cholesterol(Muchiri[5]); M: Mean, SD: Standard Deviation, SE: Standard Error

For peer review only

	Baseline M(SD)		Follow-up (6 months post-intervention) M(SE)	
	**Original (mmol/L)**	**Converted (mg/dl)**	**Original (mmol/L)**	**Converted (mg/dl)**
**Experimental Intervention**	1.10 (0.27)	42.54 (10.44)	0.99 (0.02)	38.61 (0.78)
**Control intervention**	1.10 (0.27)	42.54 (10.44)	1.0 (0.03)	39 (1.17)

Table 3: Unit transformation for HDL lipoprotein-cholesterol (Muchiri[5]); M: Mean, SD: Standard deviation, SE: Standard Error

Triglycerides (Table 4): The two studies that included biomarkers of diabetes complications used different units. Therefore, values in mmol/l were converted to mg/dl using the following formula: mg/dL = 88.57*mmol/l[4].

	Baseline M(CI)		Follow-up (6 months post-intervention) M(CI)	
	**Original (mmol/L)**	**Converted (mg/dl)**	**Original (mmol/L)**	**Converted (mg/dl)**
**Experimental Intervention**	1.40 (1.10 to 1.60)	124 (97.43 to 141.71)	1.30 (1.20 to 1.60)	115.7 (97.90 to 142.40)
**Control intervention**	1.40 (1.20 to 1.60)	124 (106.28 to 141.71)	1.50 (1.30 to 1.80)	133.5 (115.70 to 160.20)

Table 4: Unit transformation for triglycerides (Muchiri[5]); M: Mean, CI: 95% Confidence Interval

Standard Error/Standard Deviation (Tables 5-11): reporting by Muchiri[5] included standard deviation for baseline values, but standard error for follow-up values.

	Baseline		Follow-up (6 months post-intervention)	
	**Original** M(SD)	**Converted** M(SD)	**Original** M(SE)	**Converted** M(SD)
**Experimental Intervention**	10.80 (1.80)	NA	9.67 (0.29)	9.67 (1.86)
**Control intervention**	11.40 (2.20)	NA	10.3 (0.29)	10.3 (1.9)

Table 5: Transformation from Standard Error to Standard Deviation at follow-up measures of HbA1c. NA = Not applicable.

	Baseline		Follow-up (6 months post-intervention)	
	**Original**	**Converted**	**Original**	**Converted**

For peer review only

	M(SD)	M(SD)	M(SE)	M(SD)
**Experimental Intervention**	31.50 (7.00)	NA	30.50 (0.20)	30.5 (1.30)
**Control intervention**	30.40 (6.80)	NA	30.80 (0.20)	30.80 (1.28)

Table 6: Transformation from Standard Error to Standard Deviation at follow-up measures of BMI. NA = Not applicable.

	**Baseline**		**Follow-up (6 months post-intervention)**	
	**Original** M(SD)	**Converted** M(SD)	**Original** M(SE)	**Converted** M(SD)
**Experimental Intervention**	3.0 (0.90)	NA	103.74(3.12)	103.74(19.98)
**Control intervention**	3.1 (0.90)	NA	106.86 (3.12)	106.86 (19.98)

Table 7: Transformation from Standard Error to Standard Deviation at follow-up measures of LDL-Cholesterol. NA = Not applicable.

	**Baseline**		**Follow-up (6 months post-intervention)**	
	**Original** M(SD)	**Converted** M(SD)	**Original** M(SE)	**Converted** M(SD)
**Experimental Intervention**	3.0 (0.90)	NA	38.61 (0.78)	38.61 (4.99)
**Control intervention**	3.10 (0.90)	NA	39 (1.17)	39 (7.49)

Table 8: Transformation from Standard Error to Standard Deviation at follow-up measures of HDL-Cholesterol. NA = Not applicable.

	**Baseline**		**Follow-up (6 months post-intervention)**	
	**Original** M(SD)	**Converted** M(SD)	**Original** M(SE)	**Converted** M(SD)
**Experimental Intervention**	1.40 (1.10 to 1.60)	NA	115.7 (97.90 to 142.40)	115.7 (72.69)
**Control intervention**	1.40 (1.20 to 1.60)	NA	133.5 (115.70 to 160.20)	133.5 (72.69)

Table 9: Transformation from Confidence Interval (CI to Standard Deviation at follow-up measures of triglycerides. NA = Not applicable.

	**Baseline**		**Follow-up (6 months post-intervention)**	
	**Original** M(SD)	**Converted** M(SD)	**Original** M(SE)	**Converted** M(SD)
**Experimental Intervention**	142.9(22.9)	NA	134.1 (2.9)	134.1 (18.57)
**Control intervention**	143.3 (28)	NA	130.1 (2.9)	130.1 (18.57)

For peer review only

Table 10: Transformation from Standard Error to Standard Deviation at follow-up measures of systolic blood pressure. NA = Not applicable.

	**Baseline**		**Follow-up (6 months post-intervention)**	
	**Original** M(SD)	**Converted** M(SD)	**Original** M(SE)	**Converted** M(SD)
**Experimental Intervention**	84.3 (11.7)	NA	78.9 (1.5)	78.9 (9.60)
**Control intervention**	84.5 (11.7)	NA	78.8 (1.5)	78.8 (9.60)

Table 11: Transformation from Standard Error to Standard Deviation at follow-up measures of diastolic blood pressure. NA = Not applicable.

For peer review only

References

1 Ramadas A, Chan CKY, Oldenburg B, *et al.* Randomised-controlled trial of a web-based dietary intervention for patients with type 2 diabetes: Changes in health cognitions and glycemic control. *BMC Public Health* 2018;**18**:1–13. doi:10.1186/s12889-018-5640-1

2 Somogyi M. STUDIES OF ARTERIOVENOUS DIFFERENCES IN BLOOD SUGAR. *J Biol Chem* 1948;**174**:189–200. doi:10.1016/S0021-9258(18)57386-5

3 American Diabetes Association. Glycemic targets. *Diabetes Care* 2015;**38**:S33–40. doi:10.2337/dc15-S009

4 Rugge B, Balshem H, Sehgal R, *et al.* Lipid Conversion Factors. Published Online First: 2011.https://www.ncbi.nlm.nih.gov/books/NBK83505/ (accessed 13 May 2021).

5 Muchiri JW, Gericke GJ, Rheeder P. Effect of a nutrition education programme on clinical status and dietary behaviours of adults with type 2 diabetes in a resource-limited setting in South Africa: A randomised controlled trial. *Public Health Nutr* 2016;**19**:142–55. doi:10.1017/S1368980015000956

SUPPLEMENTARY MATERIAL 3

Title: Short-term effectiveness of nutritional therapy to treat type 2 diabetes in low- and middle-income countries: systematic review of randomized-controlled trials.

*Elizabeth Guilbert[1,2], Rachel Perry[3], Alex Whitmarsh[3], Sarah Sauchelli[3]**

Characteristics of included studies

For peer review only

Trial (year)	Population: age group; diagnosis; geographical setting; care setting	Mean age (years: intervention/ control); % female	Intervention; duration	Comparator	Timing of assessment	Outcomes assessed
**Askari (2018)**[39]	Age>18y; T2DM; Iran (UMIC); diabetes centre.	66/67; 65% female	Face-to-face training in nutrition and jogging based on the Belief, Attitude, Subjective, Norm and Enabling Factors (BASNEF) model; 8 sessions delivered over 4 weeks.	Standard care	Baseline, 3 months after intervention completion	HbA1c, fasting blood glucose<br>LDL and HDL lipoprotein-cholesterol, triglycerides
**Muchiri (2016)**[36]	Age>18y; T2DM; South Africa (UMIC); diabetic clinics in 2 community health centres.	59/58; 87% female	Face-to-face group tutorials and 6 follow-up sessions on diabetes diet, meal planning, portion control and gardening to improve vegetable intake, as well as a pamphlet and poster: 8 weeks.	Standard care. Pamphlet and poster	Baseline, at intervention completion, 6 and 12 months after intervention completion.	HbA1c<br>BMI, total, LDL and HDL lipoprotein-cholesterol, triglycerides Systolic blood pressure, diastolic blood pressure<br>Energy intake, % of intake from carbohydrate, protein, fat, alcohol, SFA, PUFA and MUFA, servings of vegetables, fruit, starchy foods.
**Ramadas (2018)**[37]	Age>18y; T2DM; Malaysia	50/52; 39.8% female	Personalized online dietary lessons (myDIDeA) targeted to the individual's current Dietary	Standard care	Baseline, at intervention completion, 6	HbA1c, fasting blood glucose

	(UMIC); medical or diabetic clinics at 3 public hospitals.		Stage of Change and provision of recommendations to increase fruit and vegetable consumption, staying healthy and reducing sugar intake; 12 sessions.		months after intervention completion.	DKAB questionnaire (knowledge, attitude, and behaviour score).
**Salahshouri (2018)**[38]	Age>18y; T2DM; Iran (UMIC); diabetic clinics at healthcare centres.	56/55; 69.3% female	Face-to-face group sessions based on psychological factors which determine nutrition such as avoiding temptation, communication skills training, replacing irrational thinking, religious laws around health and focusing on success; 8 weeks.	Standard care	Baseline, 3 months after intervention completion.	HbA1c, fasting blood glucose 4-part questionnaire comprising: a) demographic characteristics, b) nutritional perceptions and beliefs, c) fears, concerns, and discomfort as about nutritional diet, d) Perceived Dietary Adherence Questionnaire.

For peer review only

SUPPLEMENTARY MATERIAL 4

Title: Short-term effectiveness of nutritional therapy to treat type 2 diabetes in low- and middle-income countries: systematic review of randomized-controlled trials.

*Elizabeth Guilbert[1,2], Rachel Perry[3], Alex Whitmarsh[3], Sarah Sauchelli[3]**

Summary of findings of secondary outcomes.

Outcome	Endpoint	Participants (trials)	Effect estimate (95% CI)	GRADE
**Psychological effect: Perceived adherence to a healthy diet**	3 months	145 (1 trial)	MD 17.56(14.66 to 20.46)	Very Low
**Psychological effect: DKAB total score**	6 months	128(1 trial)	MD 5.18 (2.05 to 8.31)	Very Low
**LDL lipoprotein-cholesterol**	3 months	108 (1 trial)	MD -4.62 (-9.55 to 0.31)	Very Low
	6 months	82 (1 trial)	MD -3.12 (-11.77 to 5.53)	Very Low
**HDL lipoprotein-cholesterol**	3 months	108 (1 trial)	MD 5.79 (2.42 to 9.16)	Very Low
	6 months	82 (1 trial)	MD -0.39 (-3.15 to 2.37)	Very Low
**Triglycerides**	3 months	108 (1 trial)	MD -9.70 (-22.10 to 2.70)	Very Low
	6 months	82 (1 trial)	MD -17.80 (-49.27 to 13.67)	Very Low
**BMI**	6 months	82 (1 trial)	MD -0.30 (-0.85 to 0.25)	Very Low
**Systolic blood pressure**	6 months	82 (1 trial)	MD 4 (-4.04 to 12.04)	Very Low
**Diastolic blood pressure**	6 months	82 (1 trial)	MD 0.10 (-4.06 to 4.26)	Very Low

For peer review only

SUPPLEMENTARY MATERIAL 5

Title: Short-term effectiveness of nutritional therapy to treat type 2 diabetes in low- and middle-income countries: systematic review of randomized-controlled trials.

*Elizabeth Guilbert[1,2], Rachel Perry[3], Alex Whitmarsh[3], Sarah Sauchelli[3]**

PRISMA Checklist

Section/topic	#	Checklist Item	Reported on page #
**TITLE**			
Title	1	Identify the report as a systematic review, meta-analysis, or both.	1
**ABSTRACT**			
Structured summary	2	Provide a structured summary including, as applicable: background; objectives; data sources; study eligibility criteria, participants, and interventions; study appraisal and synthesis methods; results; limitations; conclusions and implications of key findings; systematic review registration number.	2
**INTRODUCTION**			
Rationale	3	Describe the rationale for the review in the context of what is already known.	4-5
Objectives	4	Provide an explicit statement of questions being addressed with reference to participants, interventions, comparisons, outcomes, and study design (PICOS).	4-5
**METHODS**			
Protocol and registration	5	Indicate if a review protocol exists, if and where it can be accessed (e.g., Web address), and, if available, provide registration information including registration number.	5
Eligibility criteria	6	Specify study characteristics (e.g., PICOS, length of follow-up) and report characteristics (e.g., years considered, language, publication status) used as criteria for eligibility, giving rationale.	5-6
Information sources	7	Describe all information sources (e.g., databases with dates of coverage, contact with study authors to identify additional studies) in the search and date last searched.	5
Search	8	Present full electronic search strategy for at least one database, including any limits used, such that it could be repeated.	5, Sup.Mat. 1
Study selection	9	State the process for selecting studies (i.e., screening, eligibility, included in systematic review, and, if applicable, included in the meta-analysis).	6
Data collection process	10	Describe method of data extraction from reports (e.g., piloted forms, independently, in duplicate) and any processes for obtaining and confirming data from investigators.	6-7

For peer review

Data items	11	List and define all variables for which data were sought (e.g., PICOS, funding sources) and any assumptions and simplifications made.	9, 11
Risk of bias in individual studies	12	Describe methods used for assessing risk of bias of individual studies (including specification of whether this was done at the study or outcome level), and how this information is to be used in any data synthesis.	5-7
Summary of measures	13	State the principal summary measures (e.g., risk ratio, difference in means).	6-7
Synthesis of results	14	Describe the methods of handling data and combining results of studies, if done, including measures of consistency (e.g., I2) for each meta-analysis.	6-7
Risk of bias across studies	15	Specify any assessment of risk of bias that may affect the cumulative evidence (e.g., publication bias, selective reporting within studies).	6
Additional analyses	16	Describe methods of additional analyses (e.g., sensitivity or subgroup analyses, meta-regression), if done, indicating which were pre-specified.	6-7
**RESULTS**			
Study selection	17	Give numbers of studies screened, assessed for eligibility, and included in the review, with reasons for exclusions at each stage, ideally with a flow diagram.	7
Study characteristics	18	For each study, present characteristics for which data were extracted (e.g., study size, PICOS, follow-up period) and provide the citations.	7-8
Risk of bias within studies	19	Present data on risk of bias of each study and, if available, any outcome level assessment (see item 12).	8-11
Results of individual studies	20	For all outcomes considered (benefits or harms), present, for each study: (a) simple summary data for each intervention group (b) effect estimates and confidence intervals, ideally with a forest plot.	7-8
Synthesis of results	21	Present results of each meta-analysis done, including confidence intervals and measures of consistency.	8,11
Risk of bias across studies	22	Present results of any assessment of risk of bias across studies (see Item 15).	9-11
Additional analysis	23	Give results of additional analyses, if done (e.g., sensitivity or subgroup analyses, meta-regression [see Item 16]).	8
**DISCUSSION**			
Summary of evidence	24	Summarize the main findings including the strength of evidence for each main outcome; consider their relevance to key groups (e.g., healthcare providers, users, and policy makers).	11
Limitations	25	Discuss limitations at study and outcome level (e.g., risk of bias), and at review-level (e.g., incomplete retrieval of identified research, reporting bias).	11-12
Conclusions	26	Provide a general interpretation of the results in the context of other evidence, and implications for future research.	12-13
**FUNDING**			

Funding	27	Describe sources of funding for the systematic review and other support (e.g., supply of data); role of funders for the systematic review.	16

For peer review only

BMJ Open

Short-term effectiveness of nutrition therapy to treat type 2 diabetes in low- and middle-income countries: systematic review and meta-analysis of randomised-controlled trials.

Journal:	*BMJ Open*
Manuscript ID	bmjopen-2021-056108.R1
Article Type:	Original research
Date Submitted by the Author:	15-Dec-2021
Complete List of Authors:	Guilbert, Elizabeth; University of Bristol, Faculty of Health Sciences<br>Perry, Rachel; University Hospitals Bristol and Weston NHS Foundation Trust, National Institute for Health Research Bristol Biomedical Research Centre<br>Whitmarsh, Alex; University Hospitals Bristol and Weston NHS Foundation Trust<br>Sauchelli, Sarah; University Hospitals Bristol and Weston NHS Foundation Trust
**Primary Subject Heading**:	Diabetes and endocrinology
Secondary Subject Heading:	Global health
Keywords:	General diabetes < DIABETES & ENDOCRINOLOGY, NUTRITION & DIETETICS, PUBLIC HEALTH

SCHOLARONE™
Manuscripts

I, the Submitting Author has the right to grant and does grant on behalf of all authors of the Work (as defined in the below author licence), an exclusive licence and/or a non-exclusive licence for contributions from authors who are: i) UK Crown employees; ii) where BMJ has agreed a CC-BY licence shall apply, and/or iii) in accordance with the terms applicable for US Federal Government officers or employees acting as part of their official duties; on a worldwide, perpetual, irrevocable, royalty-free basis to BMJ Publishing Group Ltd ("BMJ") its licensees and where the relevant Journal is co-owned by BMJ to the co-owners of the Journal, to publish the Work in this journal and any other BMJ products and to exploit all rights, as set out in our licence.

The Submitting Author accepts and understands that any supply made under these terms is made by BMJ to the Submitting Author unless you are acting as an employee on behalf of your employer or a postgraduate student of an affiliated institution which is paying any applicable article publishing charge ("APC") for Open Access articles. Where the Submitting Author wishes to make the Work available on an Open Access basis (and intends to pay the relevant APC), the terms of reuse of such Open Access shall be governed by a Creative Commons licence – details of these licences and which Creative Commons licence will apply to this Work are set out in our licence referred to above.

Other than as permitted in any relevant BMJ Author's Self Archiving Policies, I confirm this Work has not been accepted for publication elsewhere, is not being considered for publication elsewhere and does not duplicate material already published. I confirm all authors consent to publication of this Work and authorise the granting of this licence.

For peer review only

Short-term effectiveness of nutrition therapy to treat type 2 diabetes in low- and middle-income countries: systematic review and meta-analysis of randomised-controlled trials.

*Elizabeth Guilbert[1,2], Rachel Perry[3], Alex Whitmarsh[3], Sarah Sauchelli[3]**

[1] *Queen Elizabeth University Hospital, NHS Greater Glasgow and Clyde*
[2]*Faculty of Health Sciences, University of Bristol, Bristol, United Kingdom*
[3] *National Institute for Health Research Bristol Biomedical Research Centre, University Hospitals of Bristol and Weston NHS Foundation Trust and University of Bristol, Bristol, United Kingdom.*

Corresponding author:
Sarah Sauchelli
National Institute for Health Research Bristol Biomedical Research Centre, University Hospitals of Bristol and Weston NHS Foundation Trust and University of Bristol, Bristol, United Kingdom.
ORCID iD: orcid.org/0000-0003-3620-7671
Phone: 01173421758
Email: sarah.sauchellitoran@bristol.ac.uk

Word count: 4578
Keyword: type 2 diabetes, nutrition therapy, systematic review, LMIC

For peer review only - http://bmjopen.bmj.com/site/about/guidelines.xhtml

ABSTRACT

Objectives

This review examined the evidence arising from randomised controlled trials regarding the impact of nutrition therapy on glycaemic control in people living with type 2 diabetes mellitus (T2DM) in low- and middle-income countries (LMICs).

Design

Systematic review and meta-analysis using the Grading of Recommendations Assessment, Development, and Evaluation (GRADE) Approach. PROSPERO CRD42020188435

Data Sources

MEDLINE, EMBASE, Web of Science, OpenGrey and the International Clinical Trials Registry were searched (up to the 3d of July 2020).

Eligibility Criteria

Trials were included if they evaluated nutrition therapy in adults diagnosed with type 2 diabetes mellitus, were conducted in LMICs, measured glycaemic control and the trial included a 3-month post-intervention assessment. Nutrition therapy was defined according to the American Diabetes Association recommendations.

Data extraction and synthesis

Two independent reviewers screened the database. Study characteristics and outcome data were extracted using a data collection form. Meta-analyses were conducted for glycated haemoglobin (HbA1c) and fasting blood glucose. Trials were assessed for risk of bias (Cochrane Risk-of-Bias, v2) and overall certainty of evidence (GRADE).

Results

4 trials met inclusion criteria (total n = 463), conducted in Malaysia, Iran, and South Africa. All trials focussed on nutrition education with no direct prescription or manipulation of diet. Mean differences between intervention and standard care were -0.63% (95% CI -1.47 to 0.21) for HbA1c and -13.63 mg/dl (95% CI -37.61 to 10.34) in favour of the intervention. Given the small number of eligible trials, moderate to high risk of publication bias and serious concerns regarding consistency and precision of the evidence, certainty of evidence was deemed to be very low.

Conclusions

There is a lack of well-conducted randomised controlled trials that examine the long-term impact of nutrition therapy in LMICs, preventing firm conclusions to be made on their effectiveness. Further research is essential to discover realistic, evidence-based solutions.

See the PRISMA 2020 for Abstracts checklist in Supplementary Material 1.

Funding

SS, RP, and AW are supported by the NIHR Biomedical Research Centre at University Hospitals of Bristol and Weston NHS Foundation Trust and the University of Bristol. The views expressed are

For peer review only

those of the authors and not necessarily those of the NIHR or the Department of Health and Social Care.

Article Summary: Strengths and limitations of this study

- This is the first synthesis of evidence for the effectiveness of nutrition therapy for the management of type 2 diabetes mellitus in low- to income-countries derived from randomised controlled trials that have included post-intervention assessment.
- The review was conducted following BMJ Best Practice research methods, such as the use of GRADE (Grading of Recommendations, Assessment, Development and Evaluations) to provide a transparent critical appraisal of the certainty of evidence.
- The identification of few trials that met eligibility criteria highlight the need to build capacity for high-quality and long-term research to guide context appropriate evidence-based medicine, and to consider pragmatic trials as alternative research approaches to gather evidence.
- The focus on randomised controlled trials that included post-intervention assessment signifies that research on other interventions that adopted a different study design were not captured.

For peer review only

INTRODUCTION

Type 2 diabetes mellitus (T2DM) is rapidly becoming a global pandemic, currently affecting 8.8% of the world's population [1]. It causes significant suffering for those affected, increases mortality and costs the global economy $727 billion USD per year [1,2]. Additionally, diabetes is no longer a disease of wealthy nations. Approximately 79% of the 254 million people affected live in low- to middle-income countries (LMICs)[1,3–5]. The rising prevalence of T2DM in LMICs is particularly problematic since these countries have limited resources to implement effective interventions that are sustainable over time[4,6] and to treat the chronic comorbidities linked to T2DM[7].

LMICs display the fastest increase in the contribution of the global burden of diabetes [2]. Epidemiological studies show that development and urbanization in LMICs have led to a less active lifestyle [8,9] and an increased consumption of processed foods and sugary snacks with a high glycaemic index and [3,10]. Combined with an improvement in infectious disease management and population ageing [11], societal changes have expanded the proportional contribution of diabetes to the global burden of disease [3,12]. Nutritional interventions can offer a highly cost-effective avenue to address T2DM [6].

Nutrition therapy, which promotes healthy eating patterns by targeting nutrient intake and/or portion size [13], can ameliorate glycaemic control, weight management, and delay complications of diabetes [14–17]. Good glycaemic regulation can also aid prevention of cardiovascular disease, which is the greatest cause of morbidity and mortality in T2DM [6,16]. These effects have also been observed in the absence of pharmacological agents, when people are unable to travel to healthcare facilities or when there is a lack of education and health literacy [6,10,14]. However, high quality research on T2DM prevention and management strategies has focused primarily on high-income countries [5]. Randomised controlled trials (RCTs), considered as a gold-standard for evidence-based health interventions [18], are less common in LMICs, with 78% of RCTs for non-communicable diseases recruiting participants in high-income countries [19].

Given the scarce number of trials available in many LMICs, development and implementation of interventions lacks rigorous scientific appraisal, or will often be based on the evidence gathered in HICs. Direct application of interventions tested in HICs may not be effective in LMICs, as they may not be sensitive to context-specific characteristics of communities in LMICs in terms of religion, socioeconomic status, composition of staple foods and cultural norms [7]. These factors strongly influence both prevalence and management of T2DM and hence the success of nutritional interventions [1,14,20]. In addition, assessment of outcomes following the termination of an intervention is fundamental to evaluate its effectiveness. Weight regain is frequent in people with T2DM, particularly when continuous support provided in weight loss interventions ends [21]. Measurement of glycated haemoglobin (HbA1c), the standard measure of glucose regulation, reflects the average blood glucose levels over the 2-3 months prior to measurement. Therefore, when evaluating the effects of an intervention on glycaemic control, it is essential that a 3-month follow-up assessment is in place. This is likely to be rare in LMICs, when critical analysis of evidence from high-income countries reveals that long-term follow-up is often limited to a maximum of 8-months post-intervention [17,22,23].

The purpose of this study was to systematically review RCTs carried out in LMICs assessing the short-term effectiveness of interventions focusing on nutrition therapy to improve glycaemic control in people with T2DM. Nutrition therapy was defined according to the consensus recommendations

For peer review only - http://bmjopen.bmj.com/site/about/guidelines.xhtml

presented by the American Diabetes Association for diabetes and pre-diabetes [13], where treatment entails the modification of nutrient or whole-food intake. This definition included interventions that provide individualised, diabetes-focussed management plan and ongoing monitoring, that address individual nutrition needs and that distribute positive messaging about food choices, as well as nutrition education, lifestyle intervention programmes with goal-setting, provision of tools for day-to-day meal planning. Importantly, the review will consider those interventions that have included a 3-month post-intervention assessment of HbA1c to assess the primary outcome measure of potential benefits on glycaemic control following intervention completion. We expected that the present review would add to an earlier analysis of nutrition interventions for T2DM prevention in LMICs [24], thus providing an overview of high-quality research conducted to address the management of T2DM in LMICs.

METHODS

Search strategy and selection criteria

Following the steps laid out in our protocol (PROSPERO CRD42020188435), a systematic literature search was conducted to identify RCTs carried out in LMICs as defined by the Development Assistance Committee list of recipients of Official Development Assistance published in 2018[25]. No amendments were made. Throughout this process the PRISMA Checklist was used to ensure proper reporting of the review[26]. The databases MEDLINE, EMBASE and Web of Science were searched, as well as Open Grey and the International Clinical Trials Registry Platform. The search was completed on the 3^{d} of July 2020. Search terms can broadly be divided into 4 categories: diabetes, nutrition, and diet, LMICs and RCTs (see Supplementary Material 2 for the Medline search). The references of relevant systematic reviews identified through literature searching were manually checked for further papers, as were the reference lists of included papers. Authors of unpublished clinical trials which were included based on protocols, were contacted by email to request results or manuscripts where available.

The inclusion and exclusion criteria followed the PICO framework [27]:

Types of studies:

- Published and unpublished randomised controlled trials that investigate nutrition therapy for T2DM.
- Minimum follow-up period of 12 weeks from the end of intervention.
- Full-text reports in any language.

Participants:

- Adults (≥18 years), male and female, any ethnicity.
- Residents of a low-, middle- or upper middle-income country as defined by the Development Assistance Committee list of recipients of Official Development Assistance for 2018-2020.
- Diagnosis of T2DM based on HbA1c ≥6.5%, random blood glucose test or oral glucose tolerance test ≥11.1mmol/l.
- Exclusion: prediabetes, metabolic syndrome without definitive T2DM diagnosis, type 1 diabetes, or gestational diabetes.

Interventions:

- Nutrition interventions delivered in person, in groups, through mobile/internet-based services or any other means.
- Food supplements/replacements

For peer review only - http://bmjopen.bmj.com/site/about/guidelines.xhtml

- Calorie/diet restrictions
- Lifestyle education interventions with a focus on diet.
- Exclusion: pharmacological interventions, preventative interventions

Comparators:

- No intervention/standard or minimal care/intervention that does not include nutrition therapy.

Outcomes:

- Primary: glycaemic control (including HbA1c, fasting plasma glucose, insulin sensitivity/resistance)
- Secondary:
    - Symptoms e.g., reduction in polyuria, polydipsia, fatigue
    - Diabetic complications e.g., cardiovascular events, retinopathy, diabetic foot, nephropathy, neuropathy, hypo- and hyper-glycaemia
    - Psychological effects including quality of life and enjoyment of food.
    - Adverse effects including malnutrition & economic consequences of diet.

Quantitative outcomes only, either continuous or categorical

Screening was not limited by date or language and both published and unpublished work was reviewed. In LMICs, interventions must be able to be maintained once funding and services provided by the investigators have been withdrawn. In addition, HbA1c levels reflect average blood glucose levels over the 2-3 months prior to measurement. For this reason, trials were excluded if they had less than a 12-week follow-up period after the intervention had finished. Those trials where assessment was only carried out while the intervention was ongoing were excluded. Trials of a population with gestational or type 1 diabetes were also excluded as management strategies differ from those for people with T2DM [13,28]. Trials were included when the main component was nutrition therapy (*i.e.,* diet modification) achieved by directly prescribing a diet, meal replacement or use of supplements or by encouraging change via nutrition education. Where nutrition therapy was not the most influential component expected to contribute to changes in glycaemic control (e.g. one structured nutrition education session within a two year exercise programme, a short course of meal replacement combined with long-term metformin prescription), the RCT was excluded.

The trials identified were imported into Rayyan [29] for screening by 2 independent researchers (LG and ST) after duplicates had been removed using EndNote Web. Conflicts between researchers were resolved by consensus, with the support of a third researcher when necessary (RP).

Data analysis

Based on the inclusion and exclusion criteria, 4 papers were included after full-text screening. Data were collected by LG and checked by SS using an adapted version of the Cochrane data collection form for RCTs and non-randomised studies [30]. Data on HbA1c and fasting plasma glucose were extracted. Where HbA1c and/or fasting plasma glucose levels were reported in different measuring units across papers, mean and standard deviation of results were converted to achieve a comparable set of results (*e.g.,* mmol/L to mg/dl; See Supplementary Material 3 for conversion). Other domains of extraction included study design characteristics, population, intervention, comparator, and secondary health outcomes. Outcomes extracted were the ones reporting glucose control data after completion of the intervention, which could be at 3 months or 6 months. The data extraction form included a template for Risk of Bias assessment based on version 2.0 of the Cochrane Risk-of-Bias instrument for randomised trials. Each domain was assessed to produce

For peer review only - http://bmjopen.bmj.com/site/about/guidelines.xhtml

scores of “high” or “low” risk, or “some concern”. Data extraction was conducted independently and in duplicate. Where information was missing, attempts were made to obtain it from the authors.

Meta-analyses were conducted using Stata 16 (StataCorp LLC, College Station, TX) for the 2 primary outcomes (HbA1c and fasting blood glucose). Pooled mean differences were calculated with corresponding 95% confidence intervals. First, we pooled trials according to the time at which the post-intervention assessment was undertaken (3-6 months), and performed separate analyses at both 3 months and 6 months. Second, we generated an overall effect estimate. As we expected both clinical and methodological variation between eligible studies of which cause would be difficult to identify, random effect analyses were considered as most appropriate. Results were presented in the form of forest plots.

Overall certainty of evidence was rated using the GRADE (Grading of Recommendations Assessment, Development and Evaluation; [31]) approach, where RCTs begin at the highest level of certainty and are downgraded if concerns arise in one or more of the following 5 domains: risk of bias, inconsistency, indirectness, imprecision, and publication bias [32,33]. GRADE was completed independently by SS and RP, and consensus was reached with oversight from AW. Dispersion of true effect sizes was reported using the T^2 statistic, and the proportion of variance in point estimates due to variation was measured using the I^2 statistic. The χ^2 test was used for homogeneity. Adhering to recommendations in the Cochrane Handbook, an I^2 statistic of 50% to 90% was considered as substantial impact, and a score of 75% to 100% was deemed evidence for considerable inconsistency [34]. As the number of eligible trials was inferior to 10, we did not proceed with using a funnel plot to evaluate publication bias, as originally planned. Trials were scrutinized individually following recommendations by Guyatt and colleagues [31] to provide a final judgement of ‘strongly suspected’ or ‘undetected’ on the GRADEpro software tool.

Data Interpretation

There are no clear-cut thresholds to conclude that an intervention under evaluation is superior to standard care on the basis of HbA1c or fasting blood glucose. Previous work suggests that a reduction by 0.5% or 1% in HbA1c is often used by health professionals when making adjustments to therapy [35] and is beneficial for reducing cardiovascular disease risk, a patient important outcome [36]. Given that fasting blood glucose is often a secondary end point in RCTs, establishing a threshold for meaningful effect was also derived from vascular risk, which suggests a threshold value of 18 mg/l[37].

Patient and Public Involvement

No patients were involved in this research study. Informal discussions with health professionals in Cameroon as part of another project raised the need to conduct the systematic review before co-designing a novel intervention with their patients.

RESULTS

The initial literature search yielded 5,075 results. After duplicates were removed, 1,948 records remained. Title and abstract screening of these records was carried out by 2 reviewers and gave a percentage agreement of 91% and a Cohen’s kappa statistic of k=0.45 indicating moderate

For peer review only - http://bmjopen.bmj.com/site/about/guidelines.xhtml

agreement [38]. Eighteen trial protocols were identified as potentially relevant for which authors were contacted for results. Of these, 14 did not provide a response, 2 had not yet completed analysis and 2 returned a published manuscript. After conflicting decisions were resolved, 44 trials remained for full text eligibility checking, and 4 trials met inclusion criteria (see Figure 1). One of the most common reasons for exclusion was the absence of a 3 month post-intervention assessment (e.g., [39]).

The 4 included papers (Table 1) reported trials conducted in South Africa[40], Malaysia[41] and Iran[42,43], which are all upper-middle income countries (a subgroup of LMICs) according to the OECD [25]. Combined, the trials examined a sample size of 463 participants (mean study sample: 115.75, weighted standard deviation of sample size: 27.11). 234 participants had been assigned to the intervention arm, and 229 to the control arm. All trials recruited participants from medical or diabetic clinics; 2 were hospital based and 2 from community health centres.

All participants had been diagnosed with T2DM, had a weighted mean age of 57 years (SD: 6.4 years), and 63% (n=291) were female. 1 trial reported the presence of comorbidities (hypertension, dyslipidaemia, and a history of cardiovascular disease, 1 excluded those candidates with chronic conditions in addition to T2DM, and 2 trials did not provide information on comorbidity. The baseline weighted mean HbA1c of participants was 8.75% (SD: 1.38%) collected from the 4 trials, and the baseline weighted mean fasting blood glucose was of 154.73 mg/dl (SD: 5.41 mg/dl) collected from 3 trials.

All RCTs focussed on nutrition education to prompt people to make healthy dietary choices and improve portion control. There was no direct manipulation of nutrient or whole-food intake (e.g. prescribing a specific diet, meal replacement, or providing food supplements/substitutes). In 1 trial participants received a pamphlet and fridge/wall poster in line with standard care, complemented by an 8-week face-to-face group (6-8 people) nutrition education programme (dietitian-led) that targeted knowledge deficits and inappropriate dietary practices, and 6 follow-up sessions [40]. The 8 sessions covered the following topics: definition of diabetes, treatment, dietary guidelines (2 sessions), gardening to increase vegetable supply, meal planning (2 sessions) and meal preparation [40]. 1 trial provided dietary lesson plans (12 sessions, fortnightly) delivered via a website and tailored to each participant's current Dietary Stage of Change (DSOC, a system to classify an individual's readiness to change a health-related behaviour)[41]. The sessions included tailored recommendations aimed at improving diabetes knowledge and behaviour, address barriers to dietary change, and motivate participants. 1 trial delivered educational sessions delivered by a range of health specialists (e.g. dietitians, psychologists) in the meeting hall of a healthcare centre. These were aimed at addressing perceptions, beliefs, fears, concerns and discomforts towards a diabetic diet (e.g., based on psychological factors which determine nutrition such as avoiding temptation, communication skills training, replacing irrational thinking, religious laws around health and focusing on success) in the form of 8 one-hour sessions with groups of 23-24 participants [42]. The fourth trial delivered a group educational intervention over 4 weeks (2 70 minute training sessions per week) based on the Belief, Attitudes, Subjective Norm and Enabling Factors model; giving participants in the intervention arm information about diabetes, symptoms of hyper- and hypo-glycaemia, diet, use of food composition tables, partitioning and food replacement and appropriate intake of fruits, vegetables, and grains [43]. This trial was the only one to ask participants to record daily food consumption (though the use of those records could not be identified), conduct follow-up calls (2

For peer review only - http://bmjopen.bmj.com/site/about/guidelines.xhtml

For peer review only

calls), send weekly reminder text messages, and to include exercise in the form of a prescribed jogging regime [43].

The common outcome used to assess glycaemic control was HbA1c, and 3 trials additionally measured fasting blood glucose. Assessment of glycaemic control took place at baseline across all trials and 1 study at end of intervention. 2 trials reported post-intervention data on glycaemic control at 3-months, and 2 at 6-months. 1 study had an additional post-intervention assessment after 12 months[40]. Dropouts were present across all interventions. Overall, both HbA1c and fasting blood glucose were found to have unclear risk of bias in 1 study and evidence for high risk of bias in 1 (Figure 2). Low risk of bias was deemed in the measurement of the outcomes due to the standardised procedure adopted to measure glycaemic control, but 2 trials lacked sufficient details on participant allocation and the absence of a-priori protocol prevented the assessment of selective reporting bias. In 1 study[43], participants were excluded if they missed 2 sessions of training, but there was no indication on the number of participants who were therefore also withdrawn from the trial. A ‘high risk’ judgment was made on the domain complete accounting of patients.

For peer review only

For peer review only - http://bmjopen.bmj.com/site/about/guidelines.xhtml

Trial (year)	Population: age group; diagnosis; geographical setting; care setting	Mean age (years: intervention/ control); % female	Intervention; duration	Comparator	Timing of assessment	Outcomes assessed
**Askari (2018)**[43]	Age>18y; T2DM; Iran (UMIC); diabetes centre.	66/67; 65% female	Face-to-face training in diabetes, diet and the importance of exercise (8 sessions over 4 weeks) with weekly reminder texts, instructions to walk at least 3 times per week, 2 post-intervention calls and pamphlets given to family and relatives.	Standard care	Baseline, 3 months after intervention completion	HbA1c (%), fasting blood glucose (mg/dl), LDL and HDL lipoprotein-cholesterol (md/dl), triglycerides (md/dl)
**Muchiri (2016)**[40]	Age>18y; T2DM; South Africa (UMIC); diabetic clinics in 2 community health centre.	59/58; 87% female	Face-to-face group tutorials and 6 follow-up sessions on diabetes diet, meal planning, portion control and gardening to improve vegetable intake; 8 weeks.	Standard care. Pamphlet and poster	Baseline, at intervention completion, 6 and 12 months after intervention completion.	HbA1c (%), BMI (kg/m^2), total, LDL and HDL lipoprotein-cholesterol, triglycerides (mmol/l); systolic blood pressure (mmHg), diastolic blood pressure (mmHg), energy intake (kJ/d), % g/d of intake from carbohydrate, protein, fat, alcohol, SFA (% of energy), PUFA (% of energy) and MUFA (% of energy), servings of vegetables (servings/d), fruit (servings/d), starchy foods (servings/d).
**Ramadas (2018)**[41]	Age>18y; T2DM; Malaysia (UMIC); medical or diabetic clinics at 3 public hospitals.	50/52; 39.8% female	Personalised online dietary lessons tailored to the patient's status and recommendations to increase fruit and vegetable consumption, staying healthy and reducing sugar intake; 12 sessions.	Standard care	Baseline, at intervention completion, 6 months after intervention completion.	HbA1c (%), fasting blood glucose (mmol/L) DKAB questionnaire (knowledge, attitude, and behavior score).
**Salahshouri (2018)**[42]	Age>18y; T2DM; Iran (UMIC); diabetic clinics at healthcare centre.	56/55; 69.3% female	Face-to-face group sessions targeting perceptions, beliefs, concerns and discomfort around a diabetic diet; 8 weeks.	Standard care	Baseline, 3 months after intervention completion.	HbA1c (%), fasting blood glucose (assumed mg/dl), 4-part questionnaire comprising: a) demographic characteristics, b) nutrition perceptions and beliefs, c)

	fears, concerns, and discomfort as about nutrition diet, d) Perceived Dietary Adherence Questionnaire.

Table 1. Characteristics of included trials

For peer review only

For peer review only - http://bmjopen.bmj.com/site/about/guidelines.xhtml

Table 2 provides a summary of findings for primary outcomes. Figure 3 presents the pooled analysis for HbA1c and fasting blood glucose. As 2 trials measured these outcomes at 3 months after intervention completion and 2 at 6 months, pooled analyses were split by timepoint of assessment. At 3 months, the nutrition interventions were more effective than standard care (control), with the pooled mean differences at –1.11% (95% CI –1.64 to –0.59) for HbA1c and –23.57 mg/dl (95% CI –44.3 to –2.84) for fasting blood glucose. The advantage was less clear in trials with a 6-month post-intervention assessment, with pooled mean differences at -0.09% (95% CI -1.10 to 0.91) for HbA1c and 9mg/dl (95% CI -7.55 to 25.55) for fasting blood glucose. Similar uncertainty is present when examining all trials jointly, with pooled mean differences at -0.63% (95% CI -1.46 to 0.21; 4 trials, n=463, GRADE=very low) for HbA1c and -13.63mg/dl (95% CI -37.61 to 10.34; 3 trials, n=381, GRADE=very low) for fasting blood glucose. The point estimates for both HbA1c and fasting blood glucose include both the line of “no effect” and the threshold for concluding clinically meaningful difference.

Note, HbA1c provides an average measure of blood glucose over the previous 3 months, due to the lifecycle of red blood cells. Further, the effects of nutrition interventions are likely to be sustained in the early months post-intervention. We would therefore not expect the difference in timepoint assessment to have a clinically meaningful impact on the reported effects. The small number of trials that met inclusion criteria does not permit definite conclusions to be reached.

For peer review only

Table 2. Summary of findings for primary outcomes.

Outcomes	**Anticipated absolute effects*** (95% CI)		Relative effect (95% CI)	№ of participants (trials)	Certainty of the evidence (GRADE)
	**Risk with standard care**	**Risk with Nutrition therapy**			
HbA1c<br>Scale from: 0% to 100%<br>follow up: range 3 months to 6 months	The mean HbA1c ranged from **8.13-10.3%**	MD **0.63 % lower** (1.47 lower to 0.21 higher)	-	463 (4 RCTs)	⊕◯◯◯ VERY LOW [a,b,c,d]
Fasting Blood Glucose<br>follow up: range 3 months to 6 months	The mean fasting blood glucose ranged from **136.8 to 153.64 mg/dl.**	MD **13.63 mg/dl lower** (37.61 lower to 10.34 higher)	-	381 (3 RCTs)	⊕◯◯◯ VERY LOW [a,d,e,f]

***The risk in the intervention group** (and its 95% confidence interval) is based on the assumed risk in the comparison group and the **relative effect** of the intervention (and its 95% CI).

CI: Confidence interval; **MD:** Mean difference

GRADE Working Group grades of evidence
High certainty: We are very confident that the true effect lies close to that of the estimate of the effect
Moderate certainty: We are moderately confident in the effect estimate: The true effect is likely to be close to the estimate of the effect, but there is a possibility that it is substantially different
Low certainty: Our confidence in the effect estimate is limited: The true effect may be substantially different from the estimate of the effect
Very low certainty: We have very little confidence in the effect estimate: The true effect is likely to be substantially different from the estimate of effect

Table 2. Summary of findings for primary outcomes.

Outcomes	Anticipated absolute effects* (95% CI)		Relative effect (95% CI)	№ of participants (trials)	Certainty of the evidence (GRADE)
	**Risk with standard care**	**Risk with Nutrition therapy**			

Explanations

a. Rated down for the small number of trials. 2 trials additionally presented unclear or high risk in several domains: randomization process, missing outcome data, and selection of the reported result.

b. Heterogeneity: p = 0.01. Downgrading carried out because p<0.01 is considered as the threshold for consistency[44]. Small sample sizes, with an I^2 of 76%.

c. The optimal information size (OIS) is 106 (based on a power of 0.80 being considered as adequate), and the total sample size of the analysis is above this (n=463). However, rated down because the confidence intervals of 2 trials contain the null effect, and though there is no harm from the intervention, clinical judgement suggests that an absolute effect size of 0.63 is not sufficiently high to judge in favour of the nutrition intervention.

d. Rated down because of the small sample size of the trials, the inability to access some full text manuscripts that might have met the inclusion criteria, and likely existence of ongoing clinical trials that may have been missed.

e. Heterogeneity: p = 0.000. Downgrading carried out because p<0.01 is considered as the threshold for consistency [44]. Small sample sizes, with an I^2 of 92%.

f. The optimal information size (OIS) is 730, the total sample size of included trials is below this value (n=381). Further the confidence intervals do not exclude no effect (-7.55 to 25.55).

For peer review only - http://bmjopen.bmj.com/site/about/guidelines.xhtml

Secondary outcomes are presented in Table 3. Briefly, 1 study reported [42] a notable improvement in perceived adherence to a healthy diet 3 months post-intervention, whereas another study reported the intervention to generate benefits for dietary knowledge, attitudes and behaviour at 6 months [41]. Two trials further examined the impact of nutrition education on lipid profile and on body mass index (BMI), biomarkers of elevated risk of diabetes [45]. HDL lipoprotein-cholesterol appears to improve following the intervention when the post-intervention assessment occurs at 3 months [43], but this is no longer so when it occurs at 6 months after intervention end [40]. After 3-6 months after intervention completion, nutrition education does not seem to impact other markers of increased risk of diabetes complications (*e.g.,* heart disease) evaluated by the trials. However, the quality of the evidence is overall very poor (Grade = Very Low). No further information was reported on adverse events nor other pre-specified secondary outcomes.

Outcome	Endpoint	Participants (trials)	Effect estimate (95% CI)	GRADE
**Psychological effect: Perceived adherence to a healthy diet**	3 months	145 (1 trial)	MD in score 17.56 (14.66 to 20.46)	Very Low
**Psychological effect: DKAB total score**	6 months	128(1 trial)	MD in total score 5.18 (2.05 to 8.31)	Very Low
**LDL lipoprotein-cholesterol**	3 months	108 (1 trial)	MD -4.62 mg/dl (-9.55 to 0.31)	Very Low
	6 months	82 (1 trial)	MD -3.12 mg/dl (-11.77 to 5.53)	Very Low
**HDL lipoprotein-cholesterol**	3 months	108 (1 trial)	MD 5.79 md/dl (2.42 to 9.16)	Very Low
	6 months	82 (1 trial)	MD -0.39 mg/dl (-3.15 to 2.37)	Very Low
**Triglycerides**	3 months	108 (1 trial)	MD -9.70 mg/dl (-22.10 to 2.70)	Very Low
	6 months	82 (1 trial)	MD -17.80 mg/dl (-49.27 to 13.67)	Very Low
**BMI**	6 months	82 (1 trial)	MD -0.30 kg/m^2 (-0.85 to 0.25)	Very Low
**Systolic blood pressure**	6 months	82 (1 trial)	MD 4 mmHg (-4.04 to 12.04)	Very Low
**Diastolic blood pressure**	6 months	82 (1 trial)	MD 0.10 mmHg (-4.06 to 4.26)	Very Low

Table 3. Table of findings for secondary outcomes.

For peer review only - http://bmjopen.bmj.com/site/about/guidelines.xhtml

Apart from grouping analyses according to timepoint of post-intervention assessment, no further subgroup analyses were conducted given the limited number of trials eligible for inclusion. Table 1 presents GRADE judgements. The certainty of evidence on the short-term benefits of nutrition education for treating type 2 diabetes mellitus in low- to middle- income countries is overall very low. This is primarily due to the small number of RCTs with at least a 3-month post-intervention assessment conducted in these countries, the inconsistency between trials, high level of imprecisions and likelihood of publication bias. Quality of evidence regarding other types of nutrition therapy could not be judged due to an absence of RCTs meeting our eligibility criteria.

Supplementary Material 1 presents the completed PRISMA checklist.

DISCUSSION

This systematic review set out to synthesize the evidence on the short-term effectiveness of nutrition therapy for glycaemic control in people living with T2DM in LMICs. Only 4 trials met the eligibility criteria. The trials focused on nutrition education, delivered via a web platform or face-to-face group training. The small number of trials, considerable heterogeneity, and concerns over the methodology and reporting in some trials indicate that evidence available from RCTs at present is of very low quality. Given the very low certainty of the evidence and point estimates, the added benefit of nutrition education on HbA1c levels or fasting blood glucose in people with T2DM living in LMICs is unclear.

Findings from this review seem to contrast positive results on the effects of nutrition therapy obtained from community-based prevention strategies and lifestyle interventions, which have shown to reduce the risk of diabetes[24,46] and improve glycaemic control [5,6,46]. The discrepancy, however, may be attributed to numerous factors. First, there is a lot of heterogeneity in the types of nutritional interventions tested in LMICs [5], possibly because they have been adapted for the target setting and resources available. As this review examined outcomes at 3 and 6 months after intervention completion (*vs* at completion) and all eligible trials focused on nutrition education (*vs* other types of nutrition therapy), comparability of findings is diminished. The emphasis on a 3 month post-intervention assessment is a strength of this review over others, as HbA1c provides an aggregate measure of the previous 3 months. Second, participants' baseline Hba1c levels in three trials were above the 10% threshold used to recommend insulin therapy initiation[47], or combining lifestyle interventions with pharmacotherapy [48], but there was no information regarding participants' use of pharmacotherapy. Third, the HbA1c levels of the control group in one study improved quite notably, which could lessen the relative effectiveness of the intervention and influenced the effect estimate. Nonetheless, the wide confidence intervals in the effect estimate indicate that the true effect may be clinically meaningful and accrual of research could provide increased confidence in estimates.

A key consideration for clinicians and policy makers is that the American Diabetes Association's definition of nutrition therapy, encompasses nutrition education and interventions that yield modification of nutrient or whole-food intake [13]. Tailoring provision of nutrition guidance to the individual, delivery by an expert in diabetes care, and ongoing monitoring to permit modification as needed have been identified as fundamental features of effective nutrition therapy in type 2 diabetes care [13]. These components of nutrition therapy were not consistently present in identified trials but could strengthen observed effectiveness of nutrition therapy. In line with guidelines generated from research in HIC [49], diabetes management may also benefit from a

For peer review only - http://bmjopen.bmj.com/site/about/guidelines.xhtml

For peer review only

multi-faceted approach, with equal importance given to nutrition, physical activity, and diabetes education; with medication administered when necessary. Within the context of the low-resourced healthcare systems found in LMICs, it may be tempting to seek cost-saving solutions. Preliminary evidence gathered through this review suggests that more comprehensive nutrition therapy and lifestyle interventions may be necessary to address the growing burden of diabetes in LMICs, particularly given that the positive impact of nutrition education on glycaemic results appears to dissipate 6 months after completion of the intervention.

A weakness of this review is that the small number of trials identified did not permit sub-group analysis by separating interventions that consisted only in nutrition education from the ones with additional components (e.g. follow-up sessions, exercise). Further, risk of bias for each outcome was assessed by considering the intervention delivered in each trial in its entirety. Although we would not expect differences in individual study risk of bias assessments based on the additional components described, future research should consider the possible impact of evaluating a multicomponent intervention (e.g. adding nutrition education to pharmacotherapy) on risk of bias.

The small number of trials identified in this review reflects consensus that there is a general absence of large experimental research around diabetes management interventions in LMICs[5,50]. While conventional RCTs are considered the gold-standard for evidence-based medicine[18], which are crucial to ensure maximization of available healthcare resources[51], the use of pragmatic trials may be more appropriate to derive evidence in LMICs[52]. Short-term interventions funded by overseas agencies rarely involve local stakeholders and the research findings are less likely to be made available locally, although they may gain more international attention when undertaken by an agency from an HIC[53]. As demonstrated by this review, adopting an RCT design may also not add value to available evidence if it is not of high standard or lacks longer term assessment. It is in countries where outcomes post-interventions are least reported where continued assessment is most critical, as sustainability is essential if it is to benefit those living in the community under investigation, and if research agendas are to be set according to local needs[54].

The trials identified in this review benefit from being carried out in 3 separate countries (Malaysia, South Africa and Iran) which vary greatly in terms of culture and diet. However, all 3 countries where the trials were run are classified in the upper-middle income country subgroup of LMICs according to DAC[25], which reflects other studies showing these countries to produce the most NCD-related literature[19]. This may be due to the high prevalence of diabetes in these countries as they progress through the epidemiological transition most rapidly and hence have more relevant literature[55]. It may also be attributable to the inequity in availability and access to medical treatment that is widespread in low-income countries, which remains a problem even as non-communicable disease research increases in LMICs as a whole[1,19,56]. Though funding for nutrition research is increasing for these countries, the majority continues to be directed towards addressing undernutrition [57].

By using a robust evidence synthesis method, including Cochrane's Risk of Bias instrument 2.0 and the GRADE system, this review demonstrates that drawing conclusions from RCTs conducted in these countries may be premature if the quality of the evidence is not carefully examined. Given the short-term nature of the interventions assessed in eligible trials, measuring HbA1c levels some time after intervention completion provides a more accurate indication of the impact of these types of interventions on glycaemic regulation. However, adopting this approach signified that the review did

For peer review only - http://bmjopen.bmj.com/site/about/guidelines.xhtml

not capture other types of interventions. Another weakness of this review is that we were unable to summarise data on actual diet and physical activity. Although neither were in the pre-registered protocol as we focused on surrogate measures of patient important outcomes (e.g. HbA1c, fasting blood glucose, and BMI), dietary behaviour and physical activity should be measured and reported by RCTs on this subject, and the information should be measured in RCTs and reported in future systematic reviews. The absence of a standardised definition for nutrition therapy poses an additional challenge. Here, we based our definition on the American Diabetes Association [13], but note that none of the eligible trials had interventions where nutrient or whole-food intake was directly manipulated, as seen in HICs[e.g.,58] nor were they delivered by registered dietitians. Growing evidence points to the important role of community health workers for health promotion in LMICs[6,24] and to adapt research designs to setting in which they are implemented[59]. Being too restrictive in eligibility criteria when synthesizing the evidence may hinder advances in evidence-guided improvements in care.

Conclusions

Very low certainty of evidence impedes conclusions to be drawn on the impact of nutrition education on glycaemic control in people with T2DM living in LMICs. Even less is known about other types of nutrition therapy if we seek outcome data at least 3 months post-intervention. As T2DM becomes a growing problem in these countries, greater efforts are needed to build capacity for high-quality, context-appropriate, and long-term research in lowest-income countries.

ETHICS STATEMENT

Not applicable/No human participants included

CONTRIBUTORSHIP STATEMENT

SS and EG conceptualized the idea and led the review process. EG generated the first draft of the manuscript, and SS further refined it to adhere to the latest guidance on evaluation of the quality of evidence (*i.e.,* GRADE). RP led the design of the review methodology. AW guided the statistical aspects of the review and was responsible for the standardization of the data. Both RP and AW edited the manuscript in accordance with their expertise. All authors approved the final version of the manuscript and agree to be accountable for all aspects of the work.

COMPETING INTERESTS

There are no competing interests. The outcome of this systematic review constitutes part of the preliminary research conducted to inform the development and assessment of interventions in Cameroon.

FUNDING

SS, RP, and AW are supported by the NIHR Biomedical Research Centre at University Hospitals of Bristol and Weston NHS Foundation Trust and the University of Bristol. The views expressed are those of the authors and not necessarily those of the NIHR or the Department of Health and Social Care.

DATA SHARING

No additional data is available beyond the information provided by articles included. Data regarding the systematic review process can be requested from the corresponding author.

For peer review only - http://bmjopen.bmj.com/site/about/guidelines.xhtml

REFERENCES

1 International Diabetes Federation. *IDF Diabetes Atlas, 9th edition*. Brussels: 2019.

2 World Health Organisation. Global report on diabetes. 2016.https://www.who.int/publications/i/item/9789241565257 (accessed 24 Apr 2020).

3 Mattei J, Malik V, Wedick NM, *et al.* Reducing the global burden of type 2 diabetes by improving the quality of staple foods: The Global Nutrition and Epidemiologic Transition Initiative. *Global Health* 2015;**11**. doi:10.1186/s12992-015-0109-9

4 Glezeva N, Chisale M, McDonald K, *et al.* Diabetes and complications of the heart in Sub-Saharan Africa: An urgent need for improved awareness, diagnostics and management. Diabetes Res. Clin. Pract. 2018;**137**:10–9. doi:10.1016/j.diabres.2017.12.019

5 Afable A, Karingula NS. Evidence based review of type 2 diabetes prevention and management in low and middle income countries. *World J Diabetes* 2016;**7**:209. doi:10.4239/wjd.v7.i10.209

6 Alaofè H, Asaolu I, Ehiri J, *et al.* Community Health Workers in Diabetes Prevention and Management in Developing Countries. Ann. Glob. Heal. 2017;**83**:661–75. doi:10.1016/j.aogh.2017.10.009

7 Checkley W, Ghannem H, Irazola V, *et al.* Management of NCD in low- And middle-income countries. Glob. Heart. 2014;**9**:431–43. doi:10.1016/j.gheart.2014.11.003

8 Ley SH, Hamdy O, Mohan V, *et al.* Prevention and management of type 2 diabetes: Dietary components and nutritional strategies. Lancet. 2014;**383**:1999–2007. doi:10.1016/S0140-6736(14)60613-9

9 Ng SW, Popkin BM. Time use and physical activity: A shift away from movement across the globe. *Obes Rev* 2012;**13**:659–80. doi:10.1111/j.1467-789X.2011.00982.x

10 Micikas M, Foster J, Weis A, *et al.* A Community Health Worker Intervention for Diabetes Self-Management Among the Tz'utujil Maya of Guatemala. *Health Promot Pract* 2015;**16**:601–8. doi:10.1177/1524839914557033

11 Weisman A, Fazli GS, Johns A, *et al.* Evolving Trends in the Epidemiology, Risk Factors, and Prevention of Type 2 Diabetes: A Review. Can. J. Cardiol. 2018;**34**:552–64. doi:10.1016/j.cjca.2018.03.002

12 Boutayeb A. The double burden of communicable and non-communicable diseases in developing countries. Trans. R. Soc. Trop. Med. Hyg. 2006;**100**:191–9. doi:10.1016/j.trstmh.2005.07.021

13 Evert AB, Dennison M, Gardner CD, *et al.* Nutrition therapy for adults with diabetes or prediabetes: A consensus report. Diabetes Care. 2019;**42**:731–54. doi:10.2337/dci19-0014

14 Mechanick JI, Marchetti AE, Apovian C, *et al.* Diabetes-specific nutrition algorithm: A transcultural program to optimize diabetes and prediabetes care. *Curr Diab Rep* 2012;**12**:180–94. doi:10.1007/s11892-012-0253-z

15 Perry HB, Zulliger R, Rogers MM. Community health workers in low-, middle-, and high-income countries: An overview of their history, recent evolution, and current effectiveness. In: *Annual Review of Public Health*. Annual Reviews Inc. 2014. 399–421. doi:10.1146/annurev-publhealth-032013-182354

16 Agee MD, Gates Z, Irwin PM. Effect of medical nutrition therapy for patients with type 2 diabetes in a low-/no-cost clinic: A propensity score-matched cohort study. *Diabetes Spectr* 2018;**31**:83–9. doi:10.2337/ds16-0077

17 Taylor R. Calorie restriction for long-term remission of type 2 diabetes. Clin. Med. J. R. Coll. Physicians London. 2019;**19**:37–42. doi:10.7861/clinmedicine.19-1-37

18 Murad MH, Asi N, Alsawas M, *et al.* New evidence pyramid. Evid. Based. Med. 2016;**21**:125–7. doi:10.1136/ebmed-2016-110401

19 Fan H, Song F. An assessment of randomized controlled trials (RCTs) for non-communicable diseases (NCDs): More and higher quality research is required in less developed countries. *Sci Rep* 2015;**5**. doi:10.1038/srep13221

For peer review only - http://bmjopen.bmj.com/site/about/guidelines.xhtml

20 Forouhi NG, Misra A, Mohan V, *et al.* Dietary and nutritional approaches for prevention and management of type 2 diabetes. *BMJ* 2018;**361**. doi:10.1136/bmj.k2234

21 Guare JC, Wing RR, Grant A. Comparison of Obese NIDDM and Nondiabetic Women: Short- and Long-Term Weight Loss. *Obes Res* 1995;**3**:329–35. doi:10.1002/j.1550-8528.1995.tb00158.x

22 Steven S, Hollingsworth KG, Al-Mrabeh A, *et al.* Very low-calorie diet and 6 months of weight stability in type 2 diabetes: Pathophysiological changes in responders and nonresponders. *Diabetes Care* 2016;**39**:808–15. doi:10.2337/dc15-1942

23 Pastors JG, Warshaw H, Daly A, *et al.* The evidence for the effectiveness of medical nutrition therapy in diabetes management. Diabetes Care. 2002;**25**:608–13. doi:10.2337/diacare.25.3.608

24 Shirinzadeh M, Afshin-Pour B, Angeles R, *et al.* The effect of community-based programs on diabetes prevention in low- and middle-income countries: A systematic review and meta-analysis. Global. Health. 2019;**15**. doi:10.1186/s12992-019-0451-4

25 Organisation for Economic Co-operation and Development. Development Assistance Committee List of Official Development Assistance Recipients: Effective for reporting on 2018, 2019 and 2020 flows. 2018. http://www.oecd.org/dac/financing-sustainable-development/development-finance-standards/DAC_List_ODA_Recipients2018to2020_flows_En.pdf: OECD (accessed 24 May 2021).

26 Moher D, Liberati A, Tetzlaff J, *et al.* Preferred reporting items for systematic reviews and meta-analyses: The PRISMA statement. PLoS Med. 2009;**6**:e1000097. doi:10.1371/journal.pmed.1000097

27 Richardson W, Wilson M, Nishikawa J, *et al.* The well-built clinical question: a key to evidence-based decisions - PubMed. *ACP J Club* 1995;**123**:A12–3.https://pubmed.ncbi.nlm.nih.gov/7582737/ (accessed 5 May 2021).

28 14. Management of diabetes in pregnancy: Standards of medical care in diabetesd2019. *Diabetes Care* 2019;**42**:S165–72. doi:10.2337/dc19-S014

29 Ouzzani M, Hammady H, Fedorowicz Z, *et al.* Rayyan-a web and mobile app for systematic reviews. *Syst Rev* 2016;**5**:210. doi:10.1186/s13643-016-0384-4

30 Higgins J, Green S. *Cochrane Handbook for Systematic Reviews of Interventions.* Version 5. 2011.

31 Guyatt G, Oxman AD, Akl EA, *et al.* GRADE guidelines: 1. Introduction - GRADE evidence profiles and summary of findings tables. *J Clin Epidemiol* 2011;**64**:383–94. doi:10.1016/j.jclinepi.2010.04.026

32 Guyatt GH, Oxman AD, Vist GE, *et al.* GRADE: An emerging consensus on rating quality of evidence and strength of recommendations. *BMJ* 2008;**336**:924–6. doi:10.1136/bmj.39489.470347.ad

33 Schünemann H, Brożek J, Guyatt G, *et al.* Handbook for Grading the Quality of Evidence And the Strength of Recommendation Using the GRADE Approach. 2013.https://gdt.gradepro.org/app/%0Ahandbook/handbook.html. (accessed 25 Oct 2020).

34 Deeks J, Higgins J, Altman D. Analyzing data and undertaking meta-analyses. In: Higgins J, S G, eds. *Cochrane handbook for systematic reviews of interventions version 5.0.0.* Chichester: : Wiley 2008.

35 Lenters-Westra E, Schindhelm RK, Bilo HJG, *et al.* Differences in interpretation of haemoglobin A1c values among diabetes care professionals. *Neth J Med* 2014;**72**:462–6.

36 Selvin E, Marinopoulos S, Berkenblit G, *et al.* Meta-analysis: Glycosylated hemoglobin and cardiovascular disease in diabetes mellitus. *Ann Intern Med* 2004;**141**. doi:10.7326/0003-4819-141-6-200409210-00007

37 Sarwar N, Gao P, Kondapally Seshasai SR, *et al.* Diabetes mellitus, fasting blood glucose concentration, and risk of vascular disease: a collaborative meta-analysis of 102 prospective

For peer review only - http://bmjopen.bmj.com/site/about/guidelines.xhtml

studies. *Lancet* 2010;**375**:2215. doi:10.1016/S0140-6736(10)60484-9

38 Landis JR, Koch GG. The Measurement of Observer Agreement for Categorical Data. *Biometrics* 1977;**33**:159. doi:10.2307/2529310

39 Safarpour P, Daneshi-Maskooni M, Vafa M, *et al.* Vitamin D supplementation improves SIRT1, Irisin, and glucose indices in overweight or obese type 2 diabetic patients: A double-blind randomized placebo-controlled clinical trial. *BMC Fam Pract* 2020;**21**:1–10. doi:10.1186/s12875-020-1096-3

40 Muchiri JW, Gericke GJ, Rheeder P. Effect of a nutrition education programme on clinical status and dietary behaviours of adults with type 2 diabetes in a resource-limited setting in South Africa: A randomised controlled trial. *Public Health Nutr* 2016;**19**:142–55. doi:10.1017/S1368980015000956

41 Ramadas A, Chan CKY, Oldenburg B, *et al.* Randomised-controlled trial of a web-based dietary intervention for patients with type 2 diabetes: Changes in health cognitions and glycemic control. *BMC Public Health* 2018;**18**:1–13. doi:10.1186/s12889-018-5640-1

42 Salahshouri A, Zamani Alavijeh F, Mahaki B, *et al.* Effectiveness of educational intervention based on psychological factors on achieving health outcomes in patients with type 2 diabetes. *Diabetol Metab Syndr* 2018;**10**:67. doi:10.1186/s13098-018-0368-8

43 Askari A, Jeihooni A, Kashfi S, *et al.* The Effect of Educational Program Based on Belief, Attitude, Subjective Norm, and Enabling Factors Model on Changing the Metabolic Indices in Elderly Patients with Type II Diabetes. *Int J Prev Med* 2018;**9**:74.

44 Guyatt GH, Oxman AD, Kunz R, *et al.* GRADE guidelines: 8. Rating the quality of evidence - Indirectness. *J Clin Epidemiol* 2011;**64**:1294–302. doi:10.1016/j.jclinepi.2011.03.017

45 Gray N, Picone G, Sloan F, *et al.* Relation between BMI and diabetes mellitus and its complications among US older adults. *South Med J* 2015;**108**:29–36. doi:10.14423/SMJ.0000000000000214

46 Gyawali B, Bloch J, Vaidya A, *et al.* Community-based interventions for prevention of Type 2 diabetes in low-and middle-income countries: a systematic review. *Health Promot Int* 2019;**34**:1218–30. doi:10.1093/heapro/day081

47 American Diabetes Association. 8. Pharmacologic Approaches to Glycemic Treatment. *Diabetes Care* 2017;**40**:S64–74. doi:10.2337/DC17-S011

48 National Institute for Health and Care Excellence. Type 2 diabetes Treatment summary. 2021.https://bnf.nice.org.uk/treatment-summary/type-2-diabetes.html (accessed 2 Dec 2021).

49 American Diabetes Association. 5. Facilitating behavior change and well-being to improve health outcomes: Standards of medical care in diabetes−2021. *Diabetes Care* 2021;**44**:S53–72. doi:10.2337/dc21-S005

50 Alemayehu C, Mitchell G, Nikles J. Barriers for conducting clinical trials in developing countries- a systematic review. Int. J. Equity Health. 2018;**17**. doi:10.1186/s12939-018-0748-6

51 Siddiqi K, Newell JN. Editorials Putting evidence into practice in low-resource settings. 2005. http://www. (accessed 12 May 2021).

52 English M, Karumbi J, Maina M, *et al.* The need for pragmatic clinical trials in low and middle income settings - taking essential neonatal interventions delivered as part of inpatient care as an illustrative example. *BMC Med* 2016;**14**:1–7. doi:10.1186/s12916-016-0556-z

53 Franzen SRP, Chandler C, Siribaddana S, *et al.* Strategies for developing sustainable health research capacity in low and middle-income countries: A prospective, qualitative study investigating the barriers and enablers to locally led clinical trial conduct in Ethiopia, Cameroon and Sri Lanka. *BMJ Open* 2017;**7**. doi:10.1136/bmjopen-2017-017246

54 Laabes EP, Desai R, Zawedde SM, *et al.* How much longer will Africa have to depend on western nations for support of its capacity-building efforts for biomedical research? *Trop Med Int Heal* 2011;**16**:258–62. doi:10.1111/j.1365-3156.2010.02709.x

55 Defo BK. Demographic, epidemiological, and health transitions: Are they relevant to

For peer review only - http://bmjopen.bmj.com/site/about/guidelines.xhtml

population health patterns in Africa? *Glob Health Action* 2014;**7**. doi:10.3402/gha.v7.22443
56 Chow CK, Ramasundarahettige C, Hu W, *et al.* Availability and affordability of essential medicines for diabetes across high-income, middle-income, and low-income countries: a prospective epidemiological study. *Lancet Diabetes Endocrinol* 2018;**6**:798–808. doi:10.1016/S2213-8587(18)30233-X
57 Perez-Escamilla R, Bermudez O, Buccini GS, *et al.* Nutrition disparities and the global burden of malnutrition. *BMJ* 2018;**361**. doi:10.1136/bmj.k2252
58 Lean ME, Leslie WS, Barnes AC, *et al.* Primary care-led weight management for remission of type 2 diabetes (DiRECT): an open-label, cluster-randomised trial. *Lancet* 2018;**391**:541–51. doi:10.1016/S0140-6736(17)33102-1
59 Bekele H, Asefa A, Getachew B, *et al.* Barriers and Strategies to Lifestyle and Dietary Pattern Interventions for Prevention and Management of TYPE-2 Diabetes in Africa, Systematic Review. J. Diabetes Res. 2020;**2020**. doi:10.1155/2020/7948712

ACKNOWLEDGEMENTS

We thank Prof. Simeon Pierre Choukem (University of Dschang, Dschang, Cameroon) for his insight on nutrition interventions in Cameroon for patients with type 2 diabetes mellitus, and for encouraging us to pursue this project.

FIGURE LEGENDS

Figure 1: PRISMA study flow.

Figure 2. Risk of bias of individual trials, by primary outcome measure.

Figure 3. Change in a) HbA1c at follow-up assessment, split according to timepoint of assessment (3- or 6-months) and overall; and b) fasting blood glucose at follow-up assessment, split according to timepoint of assessment (3- or 6-months) and overall.

For peer review only

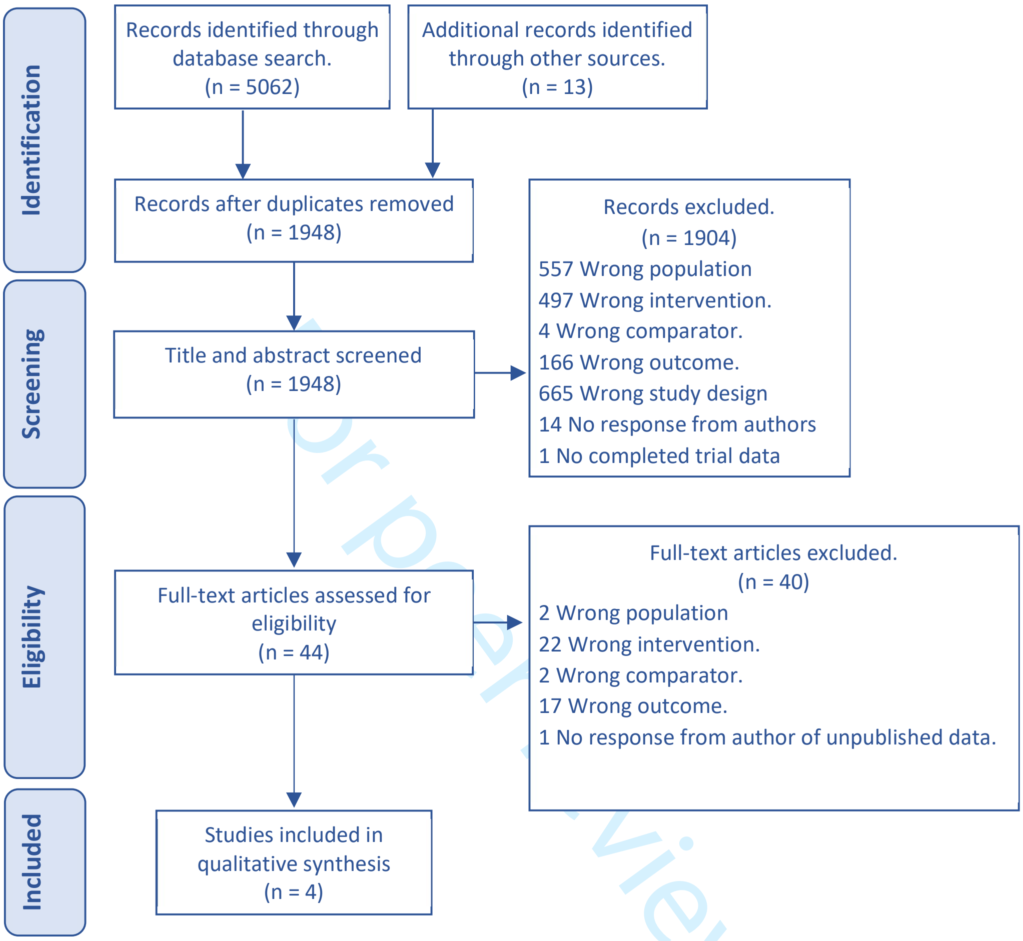
Identification
Screening
Eligibility
Included
Records identified through database search. (n = 5062)
Additional records identified through other sources. (n = 13)
Records after duplicates removed (n = 1948)
Title and abstract screened (n = 1948)
Records excluded. (n = 1904)
557 Wrong population
497 Wrong intervention.
4 Wrong comparator.
166 Wrong outcome.
665 Wrong study design
14 No response from authors
1 No completed trial data
Full-text articles assessed for eligibility (n = 44)
Full-text articles excluded. (n = 40)
2 Wrong population
22 Wrong intervention.
2 Wrong comparator.
17 Wrong outcome.
1 No response from author of unpublished data.
Studies included in qualitative synthesis (n = 4)

Outcome: HbA1c	Randomization process	Deviations from intended interventions	Missing outcome data	Measurement of the outcome	Selection of the reported result	Overall bias
Askari (2018)[39]	Some concerns	Some concerns	High risk	Low risk	Some concerns	High Risk
Muchiri (2016)[36]	Low risk	Low risk	Low risk	Low risk	Low risk	Low Risk
Ramadas (2018)[37]	Low risk	Low risk	Low risk	Low risk	Low risk	Low Risk
Salahshouri (2018)[38]	Some concerns	Low risk	Some concerns	Low risk	Some concerns	Some concerns

Outcome: Fasting blood glucose	Randomization	Deviations from intended interventions	Missing outcome data	Measurement of the outcome	Selection of the reported result	Overall bias
Askari (2018)[39]	Some concerns	Low risk	High risk	Low risk	Some concerns	High Risk
Muchiri (2016)[36]	N/A	N/A	N/A	N/A	N/A	N/A
Ramadas (2018)[37]	Low risk	Low risk	Low risk	Low risk	Low risk	Low Risk
Salahshouri (2018)[38]	Some concerns	Low risk	Some concerns	Low risk	Some concerns	Some concerns

For peer review only

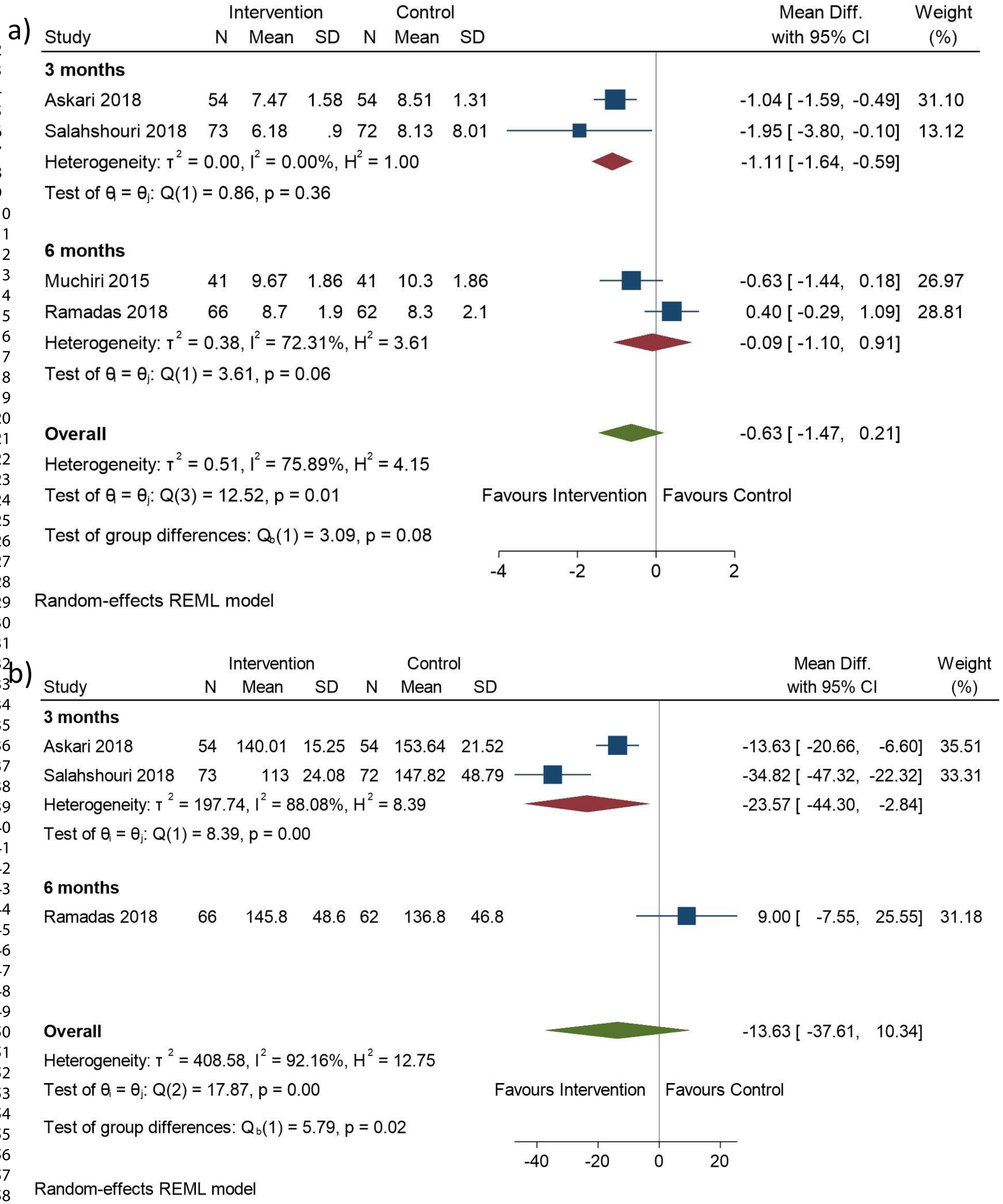
a)
Intervention
Control
Study N Mean SD N Mean SD
Mean Diff. with 95% CI
Weight (%)
3 months
Askari 2018 54 7.47 1.58 54 8.51 1.31 -1.04 [-1.59, -0.49] 31.10
Salahshouri 2018 73 6.18 .9 72 8.13 8.01 -1.95 [-3.80, -0.10] 13.12
Heterogeneity: τ² = 0.00, I² = 0.00%, H² = 1.00 -1.11 [-1.64, -0.59]
Test of θi = θj: Q(1) = 0.86, p = 0.36
6 months
Muchiri 2015 41 9.67 1.86 41 10.3 1.86 -0.63 [-1.44, 0.18] 26.97
Ramadas 2018 66 8.7 1.9 62 8.3 2.1 0.40 [-0.29, 1.09] 28.81
Heterogeneity: τ² = 0.38, I² = 72.31%, H² = 3.61 -0.09 [-1.10, 0.91]
Test of θi = θj: Q(1) = 3.61, p = 0.06
Overall -0.63 [-1.47, 0.21]
Heterogeneity: τ² = 0.51, I² = 75.89%, H² = 4.15
Test of θi = θj: Q(3) = 12.52, p = 0.01
Favours Intervention
Favours Control
Test of group differences: Qb(1) = 3.09, p = 0.08
-4
-2
0
2
Random-effects REML model
b)
Intervention
Control
Study N Mean SD N Mean SD
Mean Diff. with 95% CI
Weight (%)
3 months
Askari 2018 54 140.01 15.25 54 153.64 21.52 -13.63 [-20.66, -6.60] 35.51
Salahshouri 2018 73 113 24.08 72 147.82 48.79 -34.82 [-47.32, -22.32] 33.31
Heterogeneity: τ² = 197.74, I² = 88.08%, H² = 8.39 -23.57 [-44.30, -2.84]
Test of θi = θj: Q(1) = 8.39, p = 0.00
6 months
Ramadas 2018 66 145.8 48.6 62 136.8 46.8 9.00 [-7.55, 25.55] 31.18
Overall -13.63 [-37.61, 10.34]
Heterogeneity: τ² = 408.58, I² = 92.16%, H² = 12.75
Test of θi = θj: Q(2) = 17.87, p = 0.00
Favours Intervention
Favours Control
Test of group differences: Qb(1) = 5.79, p = 0.02
-40
-20
0
20
Random-effects REML model

SUPPLEMENTARY MATERIAL 1

Title: Short-term effectiveness of nutritional therapy to treat type 2 diabetes in low- and middle-income countries: systematic review of randomized-controlled trials.

*Elizabeth Guilbert[1,2], Rachel Perry[3], Alex Whitmarsh[3], Sarah Sauchelli[3]**

MEDLINE search strategy and number of results at each stage

1	diabetes mellitus/ or exp diabetes mellitus, type 2/ or exp hyperglycemia/ or exp metabolic syndrome/ (273807)
2	(diabet* adj5 treat*).tw. (64270)
3	(diabet* adj5 diagnos*).tw. (24777)
4	1 or 2 or 3 (322372)
5	exp Diet/ (261138)
6	*nutrition therapy/ or exp diet therapy/ (52679)
7	exp Nutrition Policy/ (9911)
8	exp nutrition assessment/ (13903)
9	nutrition disorders/ or exp overnutrition/ (213134)
10	((health* or wellness or weight or diet*) adj2 (treat* or Interven* or change* or improve*)).tw. (147274)
11	exp Food Labeling/ (3392)
12	5 or 6 or 7 or 8 or 9 or 10 or 11 (576981)
13	(afghan* or angola* or bangladesh* or benin* or bhutan* or "burkina fasso" or burundi* or cambodia* or "central african republic" or chad* or comoros or congo* or "democratic republic of the congo" or Djibouti* or eritrea* or ethiopia* or gambia* or guinea* or guinea-bissau or haiti* or kiribati* or "lao people's democratic republic" or lao or lesotho* or liberia* or madagasca* or malawi* or mali* or mauritania* or mozambique or myanmar or nepal* or niger or rwanda* or "sao tome and principe" or senegal* or "sierra leone" or "solomon islands" or somalia* or "south sudan" or sudan* or tanzania* or "timor-leste" or togo or tuvalu* or uganda* or vanuatu or yemen or zambia*).tw. (792163)
14	(armenia* or bolivia* or "cabo verde" or "cape verde" or cameroon* or congo* or "cote d'ivoire" or egypt* or "el salvador" or eswatini or georgia* or ghana* or guatemala* or honduras or india* or indonesia* or jordan* or kenya* or kosov* or kyrgyzstan or micronesia* or moldova* or mogolia* or morocc* or nicaragua* or nigeria* or pakistan* or "papua new guinea" or philippin* or "sri lanka" or "syrian arab republic" or syria* or tajikistan or tokelau or tunisia* or Ukrain* or uzbekistan or "viet nam" or "west bank and gaza strip" or "democratic people's republic of korea" or zimbabwe*).tw. (345506)
15	(albania* or algeria* or "antigua and barbuda" or argentin* or azerbaijan or belarus or belize or "bosnia and herzegovina" or botswana* or brazil* or china or colombia* or "cook islands" or "costa rica" or cuba* or dominica* or "dominican republic" or ecuador* or "equatorial guinea" or fiji or "former yugoslav republic of macedonia" or gabon or grenada or guyana* or iran* or iraq* or jamaica* or kazakhstan or lebanon or libya or Malaysia or Maldives or "marshall islands" or Mauritius or Mexico or Montenegro or Montserrat or namibia* or nauru or niue or palau or panama or paraguay or peru* or "saint helena" or "saint lucia" or "saint vincent and the grenadines" or samoa* or serbia* or "south africa" or suriname or thailand or tonga* or turkey or turkmenistan or venezuela* or "wallis and futuna").tw. (507391)
16	developing country.mp. or Developing Countries/ (75649)

17	((developing or less* developed or under developed or underdeveloped or middle income or low* income or deprived or poor) adj (countr* or nation or population)).tw. (75342)
18	13 or 14 or 15 or 16 or 17 (1632917)
19	randomi* controlled trial.tw. (81488)
20	controlled clinical trial.pt. (93000)
21	randomized controlled trial.pt. (479114)
22	random*.ab. (988614)
23	trial.ab. (448719)
24	19 or 20 or 21 or 22 or 23 (1388842)
25	4 and 12 and 18 and 24 (630)

For peer review only

SUPPLEMENTARY MATERIAL 2

Title: Short-term effectiveness of nutritional therapy to treat type 2 diabetes in low- and middle-income countries: systematic review of randomized-controlled trials.

*Elizabeth Guilbert[1,2], Rachel Perry[3], Alex Whitmarsh[3], Sarah Sauchelli[3]**

Where included studies differed in measuring units used for glycated hemoglobin (HbA1c), fasting blood glucose, and other biomarkers these were converted to enable comparison. The following formulas were applied:

Fasting Blood Glucose (Table 1): Three studies reported fasting blood glucose in milligrams per deciliter (mg/dL), whereas one study[1] measured fasting blood glucose in millimoles per liter (mmol/L). The formula applied for conversion was: mg/dL = 18.01*mmol/l [2,3].

	**Baseline M(SD)**		**Follow-up (6 months post-intervention) M(SD)**	
	**Original (mmol/L)**	**Converted (mg/dl)**	**Original (mmol/L)**	**Converted (mg/dl)**
**Experimental Intervention**	8.0 (3.9)	160.2 (70.2)	8.1 (2.7)	145.8 (48.6)
**Control intervention**	8.3 (2.9)	149.4 (52.2)	7.8 (2.6)	136.8 (46.8)

Table 1: Unit transformation for Fasting Blood Glucose (Ramadas[1]). M: Mean, SD: Standard Deviation

HDL and LDL lipoprotein-cholesterol (Tables 2 and 3): The two studies that included biomarkers of diabetes complications used different units. Therefore, values in mmol/l were converted to mg/dl using the following formula: mg/dL = 38.67*mmol/l [4]

	**Baseline M(SD)**		**Follow-up (6 months post-intervention) M(SE)**	
	**Original (mmol/L)**	**Converted (mg/dl)**	**Original (mmol/L)**	**Converted (mg/dl)**
**Experimental Intervention**	3.00 (0.90)	116.67 (34.80)	2.66 (0.08)	103.74(3.12)
**Control intervention**	3.10(0.90)	119.88 (34.80)	2.74 (0.08)	106.86 (3.12)

Table 2: Unit transformation for LDL lipoprotein-cholesterol(Muchiri[5]); M: Mean, SD: Standard Deviation, SE: Standard Error

For peer review only

	Baseline M(SD)		Follow-up (6 months post-intervention) M(SE)	
	**Original (mmol/L)**	**Converted (mg/dl)**	**Original (mmol/L)**	**Converted (mg/dl)**
**Experimental Intervention**	1.10 (0.27)	42.54 (10.44)	0.99 (0.02)	38.61 (0.78)
**Control intervention**	1.10 (0.27)	42.54 (10.44)	1.0 (0.03)	39 (1.17)

Table 3: Unit transformation for HDL lipoprotein-cholesterol (Muchiri[5]); M: Mean, SD: Standard deviation, SE: Standard Error

Triglycerides (Table 4): The two studies that included biomarkers of diabetes complications used different units. Therefore, values in mmol/l were converted to mg/dl using the following formula: mg/dL = 88.57*mmol/l[4].

	Baseline M(CI)		Follow-up (6 months post-intervention) M(CI)	
	**Original (mmol/L)**	**Converted (mg/dl)**	**Original (mmol/L)**	**Converted (mg/dl)**
**Experimental Intervention**	1.40 (1.10 to 1.60)	124 (97.43 to 141.71)	1.30 (1.20 to 1.60)	115.7 (97.90 to 142.40)
**Control intervention**	1.40 (1.20 to 1.60)	124 (106.28 to 141.71)	1.50 (1.30 to 1.80)	133.5 (115.70 to 160.20)

Table 4: Unit transformation for triglycerides (Muchiri[5]); M: Mean, CI: 95% Confidence Interval

Standard Error/Standard Deviation (Tables 5-11): reporting by Muchiri[5] included standard deviation for baseline values, but standard error for follow-up values.

	Baseline		Follow-up (6 months post-intervention)	
	**Original** M(SD)	**Converted** M(SD)	**Original** M(SE)	**Converted** M(SD)
**Experimental Intervention**	10.80 (1.80)	NA	9.67 (0.29)	9.67 (1.86)
**Control intervention**	11.40 (2.20)	NA	10.3 (0.29)	10.3 (1.9)

Table 5: Transformation from Standard Error to Standard Deviation at follow-up measures of HbA1c. NA = Not applicable.

	Baseline		Follow-up (6 months post-intervention)	
	**Original**	**Converted**	**Original**	**Converted**

For peer review only

	M(SD)	M(SD)	M(SE)	M(SD)
**Experimental Intervention**	31.50 (7.00)	NA	30.50 (0.20)	30.5 (1.30)
**Control intervention**	30.40 (6.80)	NA	30.80 (0.20)	30.80 (1.28)

Table 6: Transformation from Standard Error to Standard Deviation at follow-up measures of BMI. NA = Not applicable.

	**Baseline**		**Follow-up (6 months post-intervention)**	
	**Original** M(SD)	**Converted** M(SD)	**Original** M(SE)	**Converted** M(SD)
**Experimental Intervention**	3.0 (0.90)	NA	103.74(3.12)	103.74(19.98)
**Control intervention**	3.1 (0.90)	NA	106.86 (3.12)	106.86 (19.98)

Table 7: Transformation from Standard Error to Standard Deviation at follow-up measures of LDL-Cholesterol. NA = Not applicable.

	**Baseline**		**Follow-up (6 months post-intervention)**	
	**Original** M(SD)	**Converted** M(SD)	**Original** M(SE)	**Converted** M(SD)
**Experimental Intervention**	3.0 (0.90)	NA	38.61 (0.78)	38.61 (4.99)
**Control intervention**	3.10 (0.90)	NA	39 (1.17)	39 (7.49)

Table 8: Transformation from Standard Error to Standard Deviation at follow-up measures of HDL-Cholesterol. NA = Not applicable.

	**Baseline**		**Follow-up (6 months post-intervention)**	
	**Original** M(SD)	**Converted** M(SD)	**Original** M(SE)	**Converted** M(SD)
**Experimental Intervention**	1.40 (1.10 to 1.60)	NA	115.7 (97.90 to 142.40)	115.7 (72.69)
**Control intervention**	1.40 (1.20 to 1.60)	NA	133.5 (115.70 to 160.20)	133.5 (72.69)

Table 9: Transformation from Confidence Interval (CI to Standard Deviation at follow-up measures of triglycerides. NA = Not applicable.

	**Baseline**		**Follow-up (6 months post-intervention)**	
	**Original** M(SD)	**Converted** M(SD)	**Original** M(SE)	**Converted** M(SD)
**Experimental Intervention**	142.9(22.9)	NA	134.1 (2.9)	134.1 (18.57)
**Control intervention**	143.3 (28)	NA	130.1 (2.9)	130.1 (18.57)

For peer review only

Table 10: Transformation from Standard Error to Standard Deviation at follow-up measures of systolic blood pressure. NA = Not applicable.

	Baseline		Follow-up (6 months post-intervention)	
	**Original** M(SD)	**Converted** M(SD)	**Original** M(SE)	**Converted** M(SD)
**Experimental Intervention**	84.3 (11.7)	NA	78.9 (1.5)	78.9 (9.60)
**Control intervention**	84.5 (11.7)	NA	78.8 (1.5)	78.8 (9.60)

Table 11: Transformation from Standard Error to Standard Deviation at follow-up measures of diastolic blood pressure. NA = Not applicable.

References

1 Ramadas A, Chan CKY, Oldenburg B, *et al.* Randomised-controlled trial of a web-based dietary intervention for patients with type 2 diabetes: Changes in health cognitions and glycemic control. *BMC Public Health* 2018;**18**:1–13. doi:10.1186/s12889-018-5640-1

2 Somogyi M. STUDIES OF ARTERIOVENOUS DIFFERENCES IN BLOOD SUGAR. *J Biol Chem* 1948;**174**:189–200. doi:10.1016/S0021-9258(18)57386-5

3 American Diabetes Association. Glycemic targets. *Diabetes Care* 2015;**38**:S33–40. doi:10.2337/dc15-S009

4 Rugge B, Balshem H, Sehgal R, *et al.* Lipid Conversion Factors. Published Online First: 2011.https://www.ncbi.nlm.nih.gov/books/NBK83505/ (accessed 13 May 2021).

5 Muchiri JW, Gericke GJ, Rheeder P. Effect of a nutrition education programme on clinical status and dietary behaviours of adults with type 2 diabetes in a resource-limited setting in South Africa: A randomised controlled trial. *Public Health Nutr* 2016;**19**:142–55. doi:10.1017/S1368980015000956

For peer review only

SUPPLEMENTARY MATERIAL 3

Title: Short-term effectiveness of nutritional therapy to treat type 2 diabetes in low- and middle-income countries: systematic review and meta-analysis of randomized-controlled trials.

*Elizabeth Guilbert[1,2], Rachel Perry[3], Alex Whitmarsh[3], Sarah Sauchelli[3]**

2020 PRISMA Checklist

Section and Topic	Item #	Checklist item	Location where item is reported
**TITLE**			
Title	1	Identify the report as a systematic review.	1
**ABSTRACT**			
Abstract	2	See the PRISMA 2020 for Abstracts checklist.	3, Supp 3
**INTRODUCTION**			
Rationale	3	Describe the rationale for the review in the context of existing knowledge.	4-5
Objectives	4	Provide an explicit statement of the objective(s) or question(s) the review addresses.	4-5
**METHODS**			
Eligibility criteria	5	Specify the inclusion and exclusion criteria for the review and how studies were grouped for the syntheses.	5-6
Information sources	6	Specify all databases, registers, websites, organisations, reference lists and other sources searched or consulted to identify studies. Specify the date when each source was last searched or consulted.	5
Search strategy	7	Present the full search strategies for all databases, registers and websites, including any filters and limits used.	5, Supp 1
Selection process	8	Specify the methods used to decide whether a study met the inclusion criteria of the review, including how many reviewers screened each record and each report retrieved, whether they worked independently, and if applicable, details of automation tools used in the process.	6
Data collection process	9	Specify the methods used to collect data from reports, including how many reviewers collected data from each report, whether they worked independently, any processes for obtaining or confirming data from study investigators, and if applicable, details of automation tools used in the process.	6
Data items	10a	List and define all outcomes for which data were sought. Specify whether all results that were compatible with each outcome domain in each study were sought (e.g. for all measures, time points, analyses), and if not, the methods used to decide which results to collect.	6
	10b	List and define all other variables for which data were sought (e.g. participant and intervention characteristics, funding sources). Describe any	6

For peer review only

Section and Topic	Item #	Checklist item	Location where item is reported
		assumptions made about any missing or unclear information.	
Study risk of bias assessment	11	Specify the methods used to assess risk of bias in the included studies, including details of the tool(s) used, how many reviewers assessed each study and whether they worked independently, and if applicable, details of automation tools used in the process.	6-7
Effect measures	12	Specify for each outcome the effect measure(s) (e.g. risk ratio, mean difference) used in the synthesis or presentation of results.	6
Synthesis methods	13a	Describe the processes used to decide which studies were eligible for each synthesis (e.g. tabulating the study intervention characteristics and comparing against the planned groups for each synthesis (item #5)).	7
	13b	Describe any methods required to prepare the data for presentation or synthesis, such as handling of missing summary statistics, or data conversions.	6
	13c	Describe any methods used to tabulate or visually display results of individual studies and syntheses.	7
	13d	Describe any methods used to synthesize results and provide a rationale for the choice(s). If meta-analysis was performed, describe the model(s), method(s) to identify the presence and extent of statistical heterogeneity, and software package(s) used.	7
	13e	Describe any methods used to explore possible causes of heterogeneity among study results (e.g. subgroup analysis, meta-regression).	7
	13f	Describe any sensitivity analyses conducted to assess robustness of the synthesized results.	NA
Reporting bias assessment	14	Describe any methods used to assess risk of bias due to missing results in a synthesis (arising from reporting biases).	7
Certainty assessment	15	Describe any methods used to assess certainty (or confidence) in the body of evidence for an outcome.	7
**RESULTS**			
Study selection	16a	Describe the results of the search and selection process, from the number of records identified in the search to the number of studies included in the review, ideally using a flow diagram.	7-8, figure 1
	16b	Cite studies that might appear to meet the inclusion criteria, but which were excluded, and explain why they were excluded.	8
Study characteristics	17	Cite each included study and present its characteristics.	8-9
Risk of bias in studies	18	Present assessments of risk of bias for each included study.	12-16, figure 2
Results of individual studies	19	For all outcomes, present, for each study: (a) summary statistics for each group (where appropriate) and (b) an effect estimate and its precision (e.g. confidence/credible interval), ideally using structured tables or plots.	13-15, figure 3
Results of syntheses	20a	For each synthesis, briefly summarise the characteristics and risk of bias among contributing studies.	13-15
	20b	Present results of all statistical syntheses conducted. If meta-analysis was done, present for each the summary estimate and its precision (e.g. confidence/credible interval) and measures of statistical heterogeneity. If comparing groups, describe the direction of the effect.	13-15, figure 3
	20c	Present results of all investigations of possible causes of heterogeneity among study results.	NA

For peer review only

Section and Topic	Item #	Checklist item	Location where item is reported
	20d	Present results of all sensitivity analyses conducted to assess the robustness of the synthesized results.	NA
Reporting biases	21	Present assessments of risk of bias due to missing results (arising from reporting biases) for each synthesis assessed.	13-15
Certainty of evidence	22	Present assessments of certainty (or confidence) in the body of evidence for each outcome assessed.	13-15
**DISCUSSION**			
Discussion	23a	Provide a general interpretation of the results in the context of other evidence.	16
	23b	Discuss any limitations of the evidence included in the review.	17-18
	23c	Discuss any limitations of the review processes used.	17-18
	23d	Discuss implications of the results for practice, policy, and future research.	17-18
**OTHER INFORMATION**			
Registration and protocol	24a	Provide registration information for the review, including register name and registration number, or state that the review was not registered.	5
	24b	Indicate where the review protocol can be accessed, or state that a protocol was not prepared.	5
	24c	Describe and explain any amendments to information provided at registration or in the protocol.	5
Support	25	Describe sources of financial or non-financial support for the review, and the role of the funders or sponsors in the review.	18
Competing interests	26	Declare any competing interests of review authors.	18
Availability of data, code and other materials	27	Report which of the following are publicly available and where they can be found: template data collection forms; data extracted from included studies; data used for all analyses; analytic code; any other materials used in the review.	18

For peer review only

Section and Topic	Item #	Checklist item	Reported (Yes/No)
**TITLE**			
Title	1	Identify the report as a systematic review.	Yes
**BACKGROUND**			
Objectives	2	Provide an explicit statement of the main objective(s) or question(s) the review addresses.	Yes
**METHODS**			
Eligibility criteria	3	Specify the inclusion and exclusion criteria for the review.	Yes
Information sources	4	Specify the information sources (e.g. databases, registers) used to identify studies and the date when each was last searched.	Yes
Risk of bias	5	Specify the methods used to assess risk of bias in the included studies.	Yes
Synthesis of results	6	Specify the methods used to present and synthesise results.	Yes
**RESULTS**			
Included studies	7	Give the total number of included studies and participants and summarise relevant characteristics of studies.	Yes
Synthesis of results	8	Present results for main outcomes, preferably indicating the number of included studies and participants for each. If meta-analysis was done, report the summary estimate and confidence/credible interval. If comparing groups, indicate the direction of the effect (i.e. which group is favoured).	Yes
**DISCUSSION**			
Limitations of evidence	9	Provide a brief summary of the limitations of the evidence included in the review (e.g. study risk of bias, inconsistency and imprecision).	Yes
Interpretation	10	Provide a general interpretation of the results and important implications.	Yes
**OTHER**			
Funding	11	Specify the primary source of funding for the review.	Yes
Registration	12	Provide the register name and registration number.	Yes

For peer review only
